# The impact of aspirin exposure prior to intensive care unit admission on the outcomes for patients with sepsis-associated acute respiratory failure

**DOI:** 10.3389/fphar.2023.1125611

**Published:** 2023-03-01

**Authors:** Zongqing Lu, Pu Fang, Dunling Xia, Mengdie Li, Seruo Li, Yu Wang, Lin Fu, Gengyun Sun, Qinghai You

**Affiliations:** ^1^ Department of Respiratory and Critical Care Medicine, The First Affiliated Hospital of Anhui Medical University, Hefei, Anhui, China; ^2^ Department of Respiratory and Critical Care Medicine, Second Affiliated Hospital of Anhui Medical University, Hefei, Anhui, China

**Keywords:** aspirin, sepsis, acute respiratory failure, augmented inverse propensity weighted, generalized additive mixed model, multiple mediation model

## Abstract

**Objectives:** This present study aimed to infer the association between aspirin exposure prior to ICU admission and the clinical outcomes of patients with Sepsis-associated acute respiratory failure (S-ARF).

**Methods:** We obtained data from the Medical Information Mart for Intensive Care IV 2.0. Patients were divided into pre-ICU aspirin exposure group and Non-aspirin exposure group based on whether they took aspirin before ICU admission. The primary outcome is 28-day mortality. Augmented inverse propensity weighted was used to explore the average treatment effect (ATE) of the pre-ICU aspirin exposure. A generalized additive mixed model was used to analyze the longitudinal data of neutrophil to lymphocyte ratio (NLR), red cell distribution width (RDW), oxygenation index (P/F), dynamic lung compliance (Cdyn), mechanical power (MP), and mechanical power normalized to predicted body weight (WMP) in the two groups. A multiple mediation model was constructed to explore the possible mediators between pre-ICU aspirin exposure and outcomes of patients with S-ARF.

**Results:** A total of 2090 S-ARF patients were included in this study. Pre-ICU aspirin exposure decreased 28-day mortality (ATE, −0.1945, 95% confidence interval [CI], −0.2786 to −0.1103, *p* < 0.001), 60-day mortality (ATE, −0.1781, 95% Cl, −0.2647 to −0.0915, *p* < 0.001), and hospital mortality (ATE, −0.1502, 95%CI, −0.2340 to −0.0664, *p* < 0.001). In subgroup analysis, the ATE for 28-day mortality, 60-day mortality, and hospital mortality were not statistically significant in the coronary care unit group, high-dose group (over 100 mg/d), and no invasive mechanical ventilation (IMV) group. After excluding these non-beneficiaries, Cdyn and P/F ratio of the pre-ICU aspirin exposure group increased by 0.31mL/cmH_2_O (SE, 0.21, *p* = 0.016), and 0.43 mmHg (SE, 0.24, *p* = 0.041) every hour compared to that of non-aspirin exposure group after initialing IMV. The time-weighted average of NLR, Cdyn, WMP played a mediating role of 8.6%, 24.7%, and 13% of the total effects of pre-ICU aspirin exposure and 28-day mortality, respectively.

**Conclusion:** Pre-ICU aspirin exposure was associated with decreased 28-day mortality, 60-day mortality, and hospital mortality in S-ARF patients except those admitted to CCU, and those took a high-dose aspirin or did not receive IMV. The protective effect of aspirin may be mediated by a low dynamic level of NLR and a high dynamic level of Cdyn and WMP. The findings should be interpreted cautiously, given the sample size and potential for residual confounding.

## 1 Introduction

Sepsis or septic shock is a common etiology for hospitalizations and intensive care admissions, and the incidence is increasing yearly (Markwart et al., 2020). In sepsis, acute respiratory failure (ARF) is frequent and contributes to poor prognosis. In addition to direct pulmonary infection, systemic inflammatory response syndrome and acute secondary organ dysfunction induced by non-pulmonary infections have also been linked to ARF (Pham and Rubenfeld, 2017). Several evidence-based practices, such as early fluid resuscitation and lung-protective ventilation strategies, have been shown to improve patient outcomes (Acute Respiratory Distress Syndrome Network et al., 2000; Evans et al., 2021). However, no specific pharmacotherapy is available for sepsis or ARF.

Growing evidence has proposed the specific role of platelet activation in the pathophysiology of sepsis and its related complications (Wang et al., 2018). The platelet activation induced by endotoxin, adenosine diphosphate, thromboxane A2, and thrombin promotes the rapid formation of microvascular thrombosis, immune response, and subsequent inflammatory-coagulation cascade imbalances (Wang et al., 2018), leading to tissue hypoperfusion, organ injury, and, ultimately, the multi-organ dysfunction. Anatomic and physiologic properties of the respiratory system suggest that lungs are especially exposed to higher levels of pathogens, their virulence factors, and inflammatory cytokines in sepsis. Furthermore, large amounts of immature megakaryocytes along with haematopoietic are stored in the lung interstitium; the progenitors megakaryocytes derived from bone marrow can also circulate through the lungs and continually generate platelet in lung vasculature (Lefrançais et al., 2017). These phenomenons provide an ideal condition and environment for pulmonary microvascular thrombosis in sepsis and partly explain the sepsis-associated ARF (S-ARF) mechanisms. Consequently, the application of anti-platelet, anti-thrombosis medications has attracted increasing attention in recent years.

Aspirin, as a traditional non-selective cyclooxygenase (COX) inhibitor, is primarily used for the curing of rheumatic fever and the daily primary and secondary prevention of cardiovascular and cerebrovascular events. Moreover, preclinical studies have suggested that aspirin could be a potential agent for treating sepsis and pulmonary complications through antiplatelet activity, anti-inflammatory, inhibiting cyclooxygenase, suppressing bacterial growth, releasing nitric oxide, and modulating nuclear factor kappa B (NF-κB) pathway (Trzeciak et al., 2008; Eisen, 2012; Leeten et al., 2021). Unfortunately, there is a paucity of studies that aim to examine the impact of aspirin on S-ARF patients. Despite the evidence of favorable outcomes of aspirin in the prevention and treatment of acute respiratory distress syndrome (ARDS) (Boyle et al., 2015; Chen et al., 2015; O'Neal et al., 2011), these trials have inherent disadvantages. Firstly, the subject population was not well defined. Several studies pre-excluded patients with cardiovascular disease, while this population is more likely to have a chronic aspirin medication and at high risk of pulmonary complications under severe infections. Second, despite these concerns about its safety, few studies to date have explored the risk of thrombocytopenia and major gastrointestinal hemorrhage brought by aspirin in sepsis patients. Finally, and perhaps most importantly, most observational studies on the potential pharmacologic therapy for sepsis and its complications, particularly regarding the survival rate, are essentially a correlation analysis and did not analyze possible mediating effects between exposure and outcomes. It limits the possibility of deducing the key protective mechanisms and performing follow-up research.

Therefore, we conducted a real-world study to further characterize the possible benefit and safety of pre-ICU aspirin exposure in S-ARF patients. In this study, a doubly robust estimation based on an augmented inverse probability weighting approach (AIPW) was used to control relevant confounding factors and thereby make an accurate and robust estimation. In addition, we analyze the dynamic trend of several potential mediators using a generalized additive mixed model (GAMM). Finally, a multiple mediation analysis was performed to identify the direct and indirect effects of pre-ICU aspirin exposure on clinical outcomes.

## 2 Materials and methods

### 2.1 Data sources

The cohort of patients used in this present study was from MIMIC-IV 2.0, published on 12 June 2022. MIMIC-IV is an openly accessible database containing a comprehensive electronic medical record for over 40,000 patients admitted to intensive care units at the Beth Israel Deaconess Medical Center in Boston, Massachusetts, between 2008 and 2019. Compared with version 1.0, MIMIC-IV 2.0 supplemented more detailed follow-up records, interventions, and information from the online medical record system, which makes it possible to assess out-of-hospital mortality and better understand the previous health states.

After completing the Collaborative Institution Training Initiative Program Course offered by the National Institutes of Health in the United States and passing the examination to protect human study participants, we were eligible to use the MIMIC-IV database (Record ID: 38455175, 39691989).

### 2.2 Study population

All patients with S-ARF were initially included in this study. The S-ARF patients had sepsis at ICU admission, and ARF occurred within three subsequent days. Sepsis was diagnosed according to The Third International Consensus Definitions for Sepsis and Septic Shock (Sepsis-3.0) (Rhodes et al., 2016), the Sequential Organ Failure Assessment (SOFA) greater than 2 points based on confirmed or suspected infections. In contrast, the suspected infections have defined the records of empiric antibiotic therapy prior to or within 3 days after culture collection in the MIMIC-IV database. In addition, ARF was defined with the presence of at least one of the followings: 1) PaO_2_ < 60 mmHg by arterial blood gas test; 2) PaO_2_/FiO_2_ ratio (P/F) < 300 mmHg; 3) ventilatory support, including invasive mechanical ventilation (IMV), non-invasive mechanical ventilation and high flow nasal cannula. Minors (younger than 18 years old) and those discharged or died within 48 h after ICU admission were excluded. Additionally, we analyzed only the first ICU stay for patients admitted to the ICU more than once.

Patients who received any anti-platelet drugs other than aspirin, any anti-coagulation therapy, or dual anti-platelet therapy before ICU admission were excluded to avoid potential confounding factors.

### 2.3 Exposure

Based on the pharmacy_ID and generic names, MIMIC-IV 2.0 prescription drug file was used to identify the medication and dosage. We subdivided included patients into two groups according to the pre-ICU aspirin use: Pre-ICU aspirin exposure group and Non-aspirin exposure group. The route of administration must be oral, and the patients with the first aspirin medication record that occurred during ICU stay were excluded. Considering the possibility of discontinuation after trauma and surgery, the patients admitted to the Surgical Intensive Care Unit (SICU) or the Traumatic Surgery Intensive Care Unit (TSICU) were excluded. Furthermore, we also excluded the patients with a history of asthma to avoid the additional risk of acute asthma exacerbation after aspirin use.

### 2.4 Outcomes

The primary outcome of interest is 28-day ICU mortality, and the secondary outcomes are 60-day mortality, hospital mortality, ICU length of stay, thrombocytopenia, and major gastrointestinal hemorrhage. In MIMIC-IV 2.0, the maximum time of follow-up for each patient is exactly 1 year after their last hospital discharge, thus the INTIME records (ICU admission date extracted from ICUSTAYS Table in ICU modules), DISCHTIME (hospital discharge date and time extracted from ADMISSIONS Table in HOSP modules), and DOD records (the death date captured in either state or hospital death records, which extracted from PATIENTS Table in HOSP modules) can be applied to determine the mortality within 28-day, 60-day of admission in the ICU and hospital mortality. Thrombocytopenia was defined as a platelet count less than 100 × 10^9^/L. The major gastrointestinal hemorrhage was defined as compatible ICD-9 or ICD-10 diagnostic codes related to gastrointestinal hemorrhage during hospitalization and more than two units of RBC transfusion.

### 2.5 Variable extraction and covariates selection

The following data on the first day of ICU admission were extracted from the MIMIC-IV database: age, gender, race, weight, height, body mass index (BMI), ICU types, previous medication records, comorbidities, infectious sites, vital signs, lab tests, SOFA score, Simplify Acute Physiological Scores II (SAPS II) score, Charlson comorbidity index, and Glasgow Coma Score (GCS). When height was missing, the data stored in the online medical record system can be referred (OMR Table in HOSP modules). Comorbidities and infectious sites were identified using the recorded ICD-9 codes combined with ICD-10 codes. The details about the ICD-9 and ICD-10 codes for comorbidities and infectious site screening were presented in Supplementary Tables S1, S2. PostgreSQL programming (v4.21) and STATA software (v15.1) were used for data extracting and cleaning, respectively.

The possible covariates were determined by a directed acyclic graph (DAG), also known as a causal Bayesian network. The most exciting purpose of DAG is to minimize the structural confounding bias. For this reason, it has been frequently used to identify suitable sets of covariates in causal effects estimation (Textor et al., 2016; Fang and Zhang, 2022). In this study, the DAG was instructed by using DAGitty Web Application (Johannes Textor, Radboud University Nijmegen). After selecting the minimal sufficient adjustment set, age, gender, BMI, myocardial infarction (MIF), hypertension, cerebral infarction (CIF), chronic kidney disease (CKD), heart failure (HF), liver disease (LD), diabetes were included. The pre-ICU statin, angiotensin-converting enzyme inhibitors, and angiotensin receptor blockers (ACEI/ARB) medication, SOFA score, SAPS II score, septic shock, and pulmonary infections were also treated as covariates considering their substantial interference in the associations between the exposure and outcome. The final DAG showed in Supplementary Figure S1.

### 2.6 Augmented inverse probability weighted estimation

We estimated the causal effect sizes between pre-ICU aspirin exposure (T = 1) and above clinical outcomes Y) by calculating average treatment effect (ATE) (Kennedy and Small, 2015), which represented the average changes (Ex) before and after the treatment in the entire population. And the ATE was simulated from a model defined as Eq. 1.

$$\begin{array}{c} ATE=E\left[Y\left(1\right)-Y\left(0\right)\right]=E_{x}\left[E\left[Y\left(1\right)|X\right]-E\left[Y\left(0\right)|X\right]\right]\\ =E_{x}\left[E\left[Y|T=1,x\right]-E\left[Y|T=0,x\right]\right] \end{array}$$ (1)

For example, the POM1 represented the potential outcome score for aspirin group, while the POM0 for the non-aspirin group. ATE is equal to the POM1 minus POM2, where a greater than 0 means that aspirin will increased the incidence of outcome, and less than 0 means a trend of decrease.

We used the AIPW to obtain an unbiased estimate of ATE. This approach made it possible to not only model the treatment assignment T but also model the outcome Y and, in turn, adjust the effects of the confounding variables. Inverse probability weighting (IPW) and regression adjustment methods were applied for modeling treatment and outcome, respectively. Combined modeling of the treatment and outcome led to doubly robust methods (DR) that could provide unbiased estimates for the treatment effect even if one of the models was misspecified. After DR processing, the corresponding equation of ATE was changed to Eq. 2.

$$\begin{array}{c} ATE=\frac{1}{n}\overset{n}{\underset{i=1}{\sum }}\left\{\left[\frac{T_{i}Y_{i}^{F}}{\hat{e}\left(x_{i}\right)}-\frac{T_{i}-\hat{e}\left(x_{i}\right)}{\hat{e}\left(x_{i}\right)}\hat{m}\left(1,x_{i}\right)\right]-\left[\frac{\left(1-T_{i}\right)Y_{i}^{F}}{1-\hat{e}\left(x_{i}\right)}-\frac{T_{i}-\hat{e}\left(x_{i}\right)}{1-\hat{e}\left(x_{i}\right)}\hat{m}\left(0,x_{i}\right)\right]\right\}\\ =\frac{1}{n}\overset{n}{\underset{i=1}{\sum }}\left\{\hat{m}\left(1,x_{i}\right)+\frac{T_{i}\left(Y_{i}^{F}-\hat{m}\left(1,x_{i}\right)\right)}{\hat{e}\left(x_{i}\right)}-\hat{m}\left(0,x_{i}\right)-\frac{\left(1-T_{i}\right)\left(Y_{i}^{F}-\hat{m}\left(0,x_{i}\right)\right)}{1-\hat{e}\left(x_{i}\right)}\right\}\; \end{array}$$ (2)

The $\frac{T}{e\left(x\right)}+\frac{1-T}{1-e\left(x\right)}$ indicated the weight assigned for each sample, while the $\hat{m}\left(1,x_{i}\right)$ and $\hat{m}\left(0,x_{i}\right)$ respectively represented the estimations in intervention or control group by regression model. In this study, all possible covariates were adjusted in AIPW to get potential outcome means (POM) and ATE values. Sensitivity analyses were also performed to verify the stability of AIPW results using multivariate logistic regression and propensity score matching (PSM). PSM approach was performed based on a 1:1 matching ratio *via* the nearest neighbor, and a caliper width of 0.2 was used. The AIPW analysis was practiced using the CAUSALTRT procedure for SAS.

### 2.7 Statistical analysis

**FIGURE 1:**
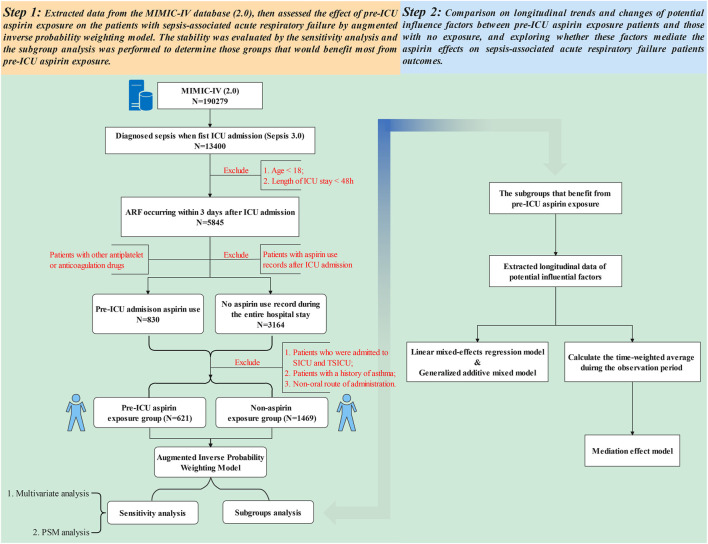
Flowchart of this present study.

The statistical study design was conceived in two steps (Figure 1). Step 1 aimed to explore the effectiveness and safety of pre-ICU aspirin exposure using AIPW, in terms of mortality, ICU length of stay, thrombocytopenia, and major gastrointestinal hemorrhage, in the included patients with S-ARF. If the pre-ICU aspirin shown to be helpful in improving prognosis in the target population, the stratification analysis within different demographic characteristics, ICU types, previous medications, comorbidities, receiving aspirins dose, and treatments during the ICU stay will be performed to identify the subgroup that may benefit most.

Step 2 aimed at exploring the underlying reasons for the prognosis improvement in the targeted beneficiary group identified in step 1. First, we extracted the longitudinal data of potential influence factors, then compared their longitudinal trajectory over time and the difference in change over time between the two groups. Secondary, to detect whether those factors mediated the protective effect of pre-ICU aspirin exposure. Since the subsequent analysis has demonstrated that pre-ICU aspirin exposure did not result in a significant improvement in mortality for patients admitted to the coronary care unit (CCU), and those who did not receive invasive mechanical ventilation (IMV) or take a high dose (over than 100 mg/d), we seem those received a low-dose (lower than 100 mg/d), IMV therapy, and not admitted in CCU as a targeted beneficiary group. Given the characteristics of this targeted beneficiary group, we selected the neutrophil-to-lymphocyte ratio (NLR), red cell distribution width (RDW), oxygenation index, dynamic lung compliance (Cdyn), mechanical power (MP), and mechanical power normalized to predicted body weight (WMP) as potential influence factors referring to the previous studies (Zhang et al., 2019; Yoo et al., 2020; Costa et al., 2021; Zhang et al., 2021). NLR and RDW were extracted as the mean values per each time frame of 1 day during the first 5 days after ICU admission, while P/F, Cdyn, MP, and WMP were also extracted as average per each 12 h time frame during the first 72 h of IMV. These calculations equation were presented in Eqs 3, 4, 5 (Serpa Neto et al., 2018; Zhang et al., 2019).

$$Cdyn=V_{T}/\left(P_{peak}-PEEP\right)$$ (3)

$$MP\left(J/\min\right)=0.098\times V_{T}\times RR\times \left(P_{peak}-\frac{1}{2}\times \left(∆P\right)\right)$$ (4)

$$WMP\left(\times 10^{-3}J/\min/kg\right)=MP/predicted\;body\;weight,$$ (5)

where, and the predicted body weight was computed by the following equations:

$$Male\left(kg\right)=50+0.91\times \left(height-152.4\right),$$

$$Female\left(kg\right)=45.5+0.91\times \left(height-152.4\right).$$

V_T_, RR, P_peak_, ΔP, and PEEP represented the tidal volume size, respiratory rate, peak pressure, driving pressure, and positive end expiratory pressure, respectively.

The longitudinal data of these factors were analyzed using the smooth curve fitting of generalized additive mixed models, consisting of generalized additive and mixed-effects models. GAMM included intercept and slope for a time as random effects to eliminate the effects of individual differences on repeated measurements. Therefore, especially appropriate when the spaced measurements are unequally and the data is unbalanced or missing (Zhang et al., 2021).

Our further attempts to assess whether the levels of NLR, RDW, P/F, MP, and WMP mediated the pre-ICU aspirin protection effect. Considering the dynamic change, the time-weighted average (TWA) for these above factors was calculated (Supplementary Figure S2) to represent the global levels during the observation time (Boyle et al., 2015). Afterward, we constructed a mediation model using PROCESS macro (v4.1) for R. Multiple mediation analyses were based on bootstrapping (10,000 bootstrap samples) using 95% confidence intervals (CI). The mediation effect was considered statistically significant if the 95% CI did not contain zero.

In the MIMIC-IV database, variables with missing data are common; however, multiple imputations were merely used to deal with missing baseline values by the “mice” package of R software. Once the missing ratio was greater than 30%, the variable was discarded. Supplementary Figure S3 showed the missing baseline values distribution and proportion before imputation. Outliers were defined as values less than the 1st percentile or greater than the 99th percentile and were winsorized using the winsor2 command in STATA software. After the Kolmogorov–Smirnov test, baseline characteristics were presented by applying descriptive statistics for both groups. Continuous variables were compared using Student's t-test or Mann-Whitney U-test, while Chi-square or Fisher’s exact test was performed in categorical variables. All statistical analyses were performed using SAS (v9.4) and R software (v3.6.2). The primary R package used in this study included mice, ggplot2, dplyr, nlme, mgcv, lme4, MatchIt, data. table, glmnet, rms, CBCgrps. A two-tailed*P*-value less than 0.05 were taken as statistically significant.

## 3 Results

### 3.1 Baseline information and clinical outcomes

In MIMIC-IV 2.0 database, a total of 13,400 patients met the Sepsis 3.0 criteria; finally, 2090 patients fulfilled the predefined inclusion criteria and were included in this study, of whom 621 patients taken aspirin prior to ICU admission. Their baseline characteristics are presented in Supplementary Table S3. Compared with the non-exposure group, patients in the pre-ICU aspirin exposure group were older, had a higher burden of pre-existing cardiac and cerebrovascular comorbidities, and were prescribed more medications. The total MV and IMV proportions were 96% (2004/2090) and 72% (1498/2090), respectively. The proportion of MV (98% vs. 95%, *p* = 0.002) and IMV (76% vs. 70%, *p* < 0.001) in the aspirin group were all significantly higher than that in the non-aspirin group. Interestingly, patients in the aspirin group had a lower percentage of 28-day mortality (20% vs. 33%, *p* < 0.001), 60-day mortality (25% vs. 37%, *p* < 0.001), and hospital mortality (19% vs. 29%, *p* < 0.001).

### 3.2 Association between pre-ICU aspirin exposure and the outcomes of sepsis-associated ARF patients

Supplementary Figure S4 presented that AIPW well resolved the inconsistencies between each included covariate. All standardized differences in the weighted column are smaller than their counterparts in the unweighted column, and all variance ratios in the weighted column are closer to 1 than their counterparts in the unweighted column.

As shown in Table 1, the ATE of aspirin on 28-day mortality was −0.1945 (95%CI, −0.2786 to −0.1103, *p* < 0.001), which means that pre-ICU aspirin exposure reduces the average 28-day mortality by 49.5% compared to non-aspirin exposure after controlling for confounders. Similarly, the ATE of −0.1781 (95%CI, −0.2647 to −0.0915, *p* < 0.001) for 60-day mortality and ATE of −0.1502 (95%CI, −0.2340 to −0.0664, *p* < 0.001) for hospital mortality, indicates that a 42.89% decrease in 60-day mortality and a 43.17% decrease in-hospital mortality in the aspirin group compared to non-aspirin group, respectively. While there is no significant difference in the length of ICU stay (ATE, 0.1462, 95%CI, −0.7857 to 1.0781, *p* = 0.759), thrombocytopenia (ATE, 0.0183, 95%CI, −0.0499 to 0.0865, *p* = 0.599), and major gastrointestinal hemorrhage risk (ATE, 0.0254, 95%CI, −0.0046 to 0.0555, *p* = 0.098).

**TABLE 1:** Augmented inverse propensity weighted analysis of causal effect of pre-ICU aspirin exposure on 28-day mortality, 60-day mortality ICU, hospital mortality, ICU length of stay, thrombocytopenia and major gastrointestinal hemorrhage risk.

Outcomes		Estimate	Robust SE	Wald 95% CI	*Z*	*P*
28-day mortality						
POM	Aspirin users	0.1884	0.0289	0.1318–0.2449	6.53	<0.001
POM	Non-users	0.3829	0.0319	0.3204–0.4453	12.02	<0.001
ATE		−0.1945	0.0429	−0.2786–−0.1103	−4.53	**<0.001**
Percentage reduction in ATE (%)		49.50				
60-day mortality						
POM	Aspirin users	0.2372	0.0308	0.1767–0.2976	7.69	<0.001
POM	Non-users	0.4152	0.0317	0.3530–0.4775	13.08	<0.001
ATE		−0.1781	0.0442	−0.2647–−0.0915	−4.03	**<0.001**
Percentage reduction in ATE (%)		42.89				
Hospital mortality						
POM	Aspirin users	0.1976	0.0288	0.1411–0.2542	6.85	<0.001
POM	Non-users	0.3479	0.0316	0.2858–0.4099	10.99	<0.001
ATE		−0.1502	0.0428	−0.2340–−0.0664	−3.51	**<0.001**
Percentage reduction in ATE (%)		43.17				
ICU length of stay						
POM	Aspirin users	7.5655	0.4132	6.7556–8.3754	18.31	<0.001
POM	Non-users	7.4193	0.2489	6.9314–7.9072	29.80	<0.001
ATE		0.1462	0.4755	−0.7857 - 1.0781	0.31	0.759
Percentage reduction in ATE (%)		−5.61				
Thrombocytopenia during ICU stay						
POM	Aspirin users	0.3811	0.0310	0.3202–0.4419	12.27	<0.001
POM	Non-users	0.3627	0.0168	0.3297–0.3958	21.53	<0.001
ATE		0.0183	0.0348	−0.0499-0.0865	0.53	0.599
Percentage reduction in ATE (%)		-				
Major gastrointestinal hemorrhage risk						
POM	Aspirin users	0.0538	0.0149	0.0246–0.0829	3.62	<0.001
POM	Non-users	0.0283	0.0039	0.0207–0.0360	7.28	<0.001
ATE		0.0254	0.0153	−0.0046-0.0555	1.66	0.098
Percentage reduction in ATE (%)		-				

Adjusted for age, gender, BMI, SOFA, SAPSII, diabetes, cerebral infarction, myocardial infarction, hypertension, coronary heart disease, heart failure, chronic kidney disease, liver disease, statin, ACEI/ARB, pulmonary infection, septic shock, Percentage reduction in ATE, was calculated as: ATE % = (POM, non-users - POM, aspirin users) *100/POM, non-users. POM: potential outcome means; ATE: average treatment effect; SE: standard error; BMI: body mass index; SOFA: the sequential organ failure assessment; SAPSII: Simplified Acute Physiology Score II. Bold font indicates that the results of ATE had significant statistical difference.

### 3.3 Subgroup analysis and sensitivity analysis

Supplementary Figures S5–S10 presented the results of subgroup analysis for each clinical outcome. We found that pre-ICU aspirin exposure had no ameliorative effect on 28-day, 60-day, and hospital mortality in the subgroup who did not receive IMV curing or taken a high-dose aspirin (>100 mg/L) and those who were admitted to CCU (Supplementary Figures S5–S7).

In addition, comparing with those who did not received heparin during ICU stay, the patients treated with aspirin plus heparin showed lower hospital mortality (ATE, −0.2848, 95%CI, −0.5408 to −0.0288, *p* = 0.0292). A shorter ICU stay was only observed in CVICU (ATE, −2.7248, 95%CI, −4.1793 to −1.2703, *p* < 0.001) and those who had previously taken ACEI/ARB (ATE, −3.5814, 95%CI, −5.2703 to −1.8925, *p* < 0.001), or used heparin during ICU stay (ATE, −1.7241, 95%CI, −2.5623 to −0.8859, *p* < 0.001) (Supplementary Figure S8). However, it is noted that the thrombocytopenia risk increased in elderly patients (ATE, 0.1337, 95%CI, 0.0503 to 0.2171, *p* = 0.0089), and those combined with septic shock (ATE, 0.0726, 95%CI, 0.0024 to 0.1426, *p* = 0.0428), received IMV (ATE, 0.1283, 95%CI, 0.0544 to 0.2023, *p* < 0.001) or heparin curing (ATE, 0.0991.95%CI, 0.0152 to 0.183, *p* = 0.0206) (Supplementary Figure S9). In addition, an increased major gastrointestinal hemorrhage risk was also found in elderly patients (ATE, 0.0456, 95%CI, 0.0114 to 0.0798, *p* = 0.0089), and those combined with thrombocytopenia (ATE, 0.1914, 95%CI, 0.0408 to 0.3421, *p* = 0.0128) or liver disease (ATE, 0.1919, 95%CI, 0.0388 to 0.345, *p* = 0.014) at ICU admission (Supplementary Figure S10).

After PSM, 486 patients, including 243 patients with pre-ICU aspirin exposure, were matched by a 1:1 matching ratio. Furthermore, the bias of all confounding factors was well resolved (Supplementary Figure S11). The sensitivity analysis demonstrated that the PSM approach and multivariate logistic regression analysis results after adjusting for confounders were all consistent with that of AIPW (Supplementary Table S4). Meanwhile, the stratification analysis also proved that the improvement in survival had disappeared in the patients who had not received IMV treatment during ICU stay, were admitted to CCU, or took a high-dose aspirin (Supplementary Table S5).

### 3.4 Effect of pre-ICU aspirin exposure on longitudinal NLR and RDW within the first 5 days after ICU admission

We selected the cohort after PSM for subsequent analysis. Based on the results of AIPW, subgroup, and sensitivity analysis, the patients who had not received IMV treatment during ICU stay, were admitted to CCU, or took a high-dose were excluded. Finally, 282 patients were selected, including 131 patients with pre-ICU aspirin exposure.

**FIGURE 2:**
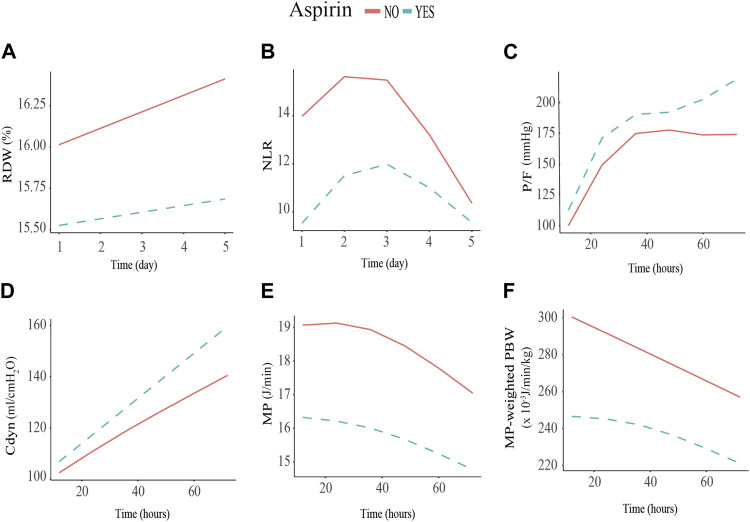
Association between red cell distribution width (RDW), neutrophil to lymphocyte ratio (NLR), P/F ratio, dynamic lung compliance (Cdyn), mechanical power (MP), and MP normalized to predicted body weight (WMP) and low-dose aspirin exposure using the generalized additive mixed model. **(A)** initial RDW level of the pre-ICU low-dose aspirin exposure group is significantly lower than that of the non-exposure group (−0.60%, SE = 0.27, *p =* 0.027); **(B)** initial NLR level of the low-dose aspirin exposure group is significantly lower than that of the non-exposure group (−3.83%, SE = 1.43, *p =* 0.008); **(C)** P/F of the low-dose aspirin exposure group increased by 0.43 mmHg (SE = 0.24, *p =* 0.041) every hour compared to that of the non-exposure group within 72 h after receiving invasive mechanical ventilation; **(D)** Cdyn of the low-dose aspirin exposure group increased by 0.31mL/cmH_2_0 (SE = 0.21, *p =* 0.016) every hour compared to that of the non-exposure group within 72 h after receiving invasive mechanical ventilation; **(E, F)** MP (−2.80J/min, SE = 0.94, *p =* 0.003) and WMP levels (−44.93 × 10^−3^ J/min/kg, SE = 9.7, *p =* 0.001) of the low-dose aspirin exposure group are significantly lower than that of the non-exposure group within the initial 12 h after receiving invasive mechanical ventilation.

For these 282 patients, the values of RDW and NLR and the corresponding comparisons between the pre-ICU aspirin exposure group and the non-aspirin group for different periods were presented in Supplementary Figure S12 and Supplementary Table S6. The GAMM model demonstrated a significant difference between the two groups in NLR (*β*, −3.83, SE, 1.43, *p* = 0.008) and RDW (*β*, −0.60, SE, 0.27, *p* = 0.027) on day 1 of ICU admission. The fitted curves showed that the levels of RDW rose linearly over time in the two groups, while the NLR levels tended to rise initially and later fall. However, there are no significant interaction effects between groups and time as either a categorical or continuous variable, indicating that pre-ICU aspirin exposure did not influence the trends for change in RDW and NLR within the first 5 days after ICU admission (Table 2; Figure 2).

**TABLE 2:** Longitudinal red cell distribution width, neutrophil to lymphocyte ratio, PaO_2_/FiO_2_, dynamic lung compliance, mechanical power, and mechanical power normalized to predicted body weight derived from a linear mixed-effects regression model.

	NLR		RDW		Cdyn		P/F		MP		WMP	
	*β(SE)*	*P*	*β(SE)*	*P*	*β(SE)*	*P*	*β(SE)*	*P*	*β(SE)*	*P*	*β(SE)*	*P*
T1 (aspirin)-T1 (non-aspirin)	−3.83 (1.43)	**0.008**	−0.60 (0.27)	**0.027**	6.69 (7.77)	0.390	15.39 (11.68)	0.189	−2.80 (0.94)	**0.003**	−44.93 (9.70)	**0.001**
^a^Group (aspirin) x T1-T2	0.97 (4.07)	0.813	0.10 (0.10)	0.242	0.51 (12.14)	0.966	18.93 (13.66)	0.167	−0.50 (1.11)	0.65	−2.68 (16.04)	0.867
^a^Group (aspirin) x T1-T3	0.33 (3.57)	0.925	−0.03 (0.09)	0.755	−1.79 (13.24)	0.892	−7.04 (14.48)	0.627	−0.37 (1.22)	0.76	4.48 (17.44)	0.797
^a^Group (aspirin) x T1-T4	1.94 (4.50)	0.668	−0.15 (0.09)	0.095	11.60 (13.82)	0.401	9.60 (15.45)	0.535	−0.29 (1.34)	0.83	11.01 (19.21)	0.566
^a^Group (aspirin) x T1-T5	3.33 (3.96)	0.402	−0.17 (0.09)	0.063	5.35 (14.57)	0.714	11.94 (16.02)	0.456	−0.50 (1.36)	0.71	11.16 (19.50)	0.567
^a^Group (aspirin) x T1-T6					20.80 (15.01)	**0.019**	33.65 (16.28)	**0.040**	−0.08 (1.43)	0.95	8.28 (20.28)	0.683
^b^Group x T	0.95 (0.84)	0.261	−0.06 (0.02)	0.420	0.31 (0.21)	**0.016**	0.43 (0.24)	**0.041**	0.0006 (0.02)	0.98	0.25 (0.29)	0.38
Observations	197		282		282		250		267		241	
Akaike Inf. Crit	331.62		320.47		282.36		297.60		319.18		268.53	
Bayesian Inf. Crit	361.48		362.03		353.9		334.82		346.34		334.24	
Log Likelihood	−105.62		−104.47		−97.18		−99.80		−105.59		−110.27	

For NLR, and RDW, T1-T5 represent the 1st, 2nd, 3rd, 4th, and 5th day after ICU, admission, while the T1-T6 represent the 12th, 24th, 36th, 48th, 60th, and 72nd hour after initiation of invasive mechanical ventilation for Cdyn, P/F, MP, and WMP. T_n_-T_m_(group A) indicates the difference between two values measured in time n) and time m) in group A, while the Group(aspirin) x T_n_-T_m_ represent the value of ([T_n_-T_m_(aspirin)] - [T_n_-T_m_(non-aspirin)]). Group x T represents the interaction test which indicates the difference trends of the above-mentioned variables between two groups over the time period measured. Bold font indicate a significant statistical difference by linear mixed-effects regression model.

^a^ time treated as a categorical variable.

^b^ time treated as a continuous variables. NLR, neutrophil to lymphocyte ratio; RDW, red cell distribution width; PF, oxygenation index; Cdyn, pulmonary dynamic compliance; MP, mechanical power.

### 3.5 Effect of pre-ICU aspirin exposure on longitudinal P/F ratio, cdyn, MP and WMP within the first 72 h after initialing IMV

For these 282 patients, the values of P/F ratio, Cdyn, MP, and WMP, and the corresponding comparisons between the pre-ICU aspirin exposure group and non-aspirin group for different periods are presented in Supplementary Figure S12 and Supplementary Table S6. The GAMM model showed that there are significantly different between the pre-ICU aspirin exposure group and non-aspirin group in MP (*β*, −2.80, SE, 0.94, *p* = 0.003) and WMP (*β*, −44.93, SE, 9.7, *p* = 0.001), within first 12 h after initialing IMV, but not Cdyn and P/F ratio. Further, there was significant group × time interaction effects in Cdyn (*β*, 20.80, SE, 15.01, *p* = 0.019) and P/F ratio (*β*, 33.65, SE, 16.28, *p* = 0.04) from 12 to 72 h. When the time was treated as a continuous variable, the interaction effect between groups and time were 0.31 (SE, 0.21, *p* = 0.016) and 0.43 (SE, 0.24, *p* = 0.041) for Cdyn and P/F ratio, respectively. It indicated that the Cdyn and P/F ratio of the pre-ICU aspirin exposure group increased by 0.31 mL/cmH_2_O and 0.43 mmHg every hour compared to the non-aspirin exposure group (Table 3; Figure 2) within the first 72 h after receiving IMV initialing. After adjusting the NLR level when initialing IMV, the trends for change in the P/F ratio, Cdyn, MP, and WMP were also similar between the two groups (Supplementary Figure S13).

**TABLE 3:** The effect size of each path in the multiple mediation analysis.

Outcome: 28-day mortality	Effect	BoostSE	95% CI	Relative mediation effect (%)
^a^Direct effect (Aspirin → Outcome)	−0.73	0.45	**−1.61, -0.14**	
^b^Indirect effect (Aspirin → TWA_NLR → Outcome)	−0.14	0.15	**−0.55, -0.04**	8.6
^c^Indirect effect (Aspirin → TWA_RDW → Outcome)	−0.16	0.12	−0.47, 0.03	9.9
^d^Indirect effect (Aspirin → TWA_PF → Outcome)	0.02	0.10	−0.24, 0.20	1.2
^e^Indirect effect (Aspirin → TWA_Cdyn → Outcome)	−0.40	0.18	**−0.67, -0.03**	24.7
^f^Indirect effect (Aspirin → TWA_WMP → Outcome)	−0.21	0.14	**−0.54, -0.10**	13.0
Total indirect effect (b + c + d + e + f)	−0.89	0.34	**−1.60, -0.37**	54.9
Outcome: Hospital mortality	Effect	BoostSE	**95% CI**	
^a^Direct effect (Aspirin → Outcome)	−0.65	0.46	**−1.41, -0.38**	
^b^Indirect effect (Aspirin → TWA_NLR → Outcome)	−0.09	0.14	**−0.64, -0.04**	6.5
^c^Indirect effect (Aspirin → TWA_RDW → Outcome)	−0.13	0.11	−0.42, 0.01	9.3
^d^Indirect effect (Aspirin → TWA_PF → Outcome)	0.02	0.10	−0.20, 0.23	1.4
^e^Indirect effect (Aspirin → TWA_Cdyn → Outcome)	−0.32	0.19	**−0.74, -0.01**	23.0
^f^Indirect effect (Aspirin → TWA_WMP → Outcome)	−0.21	0.16	**−0.65, -0.04**	15.1
Total indirect effect (b + c + d + e + f)	−0.74	0.31	**−1.51, -0.23**	53.2
Outcome: 60-day mortality	Effect	BoostSE	**95% CI**	
^a^Direct effect (Aspirin → Outcome)	−0.81	0.43	**−1.35, -0.32**	
^b^Indirect effect (Aspirin → TWA_NLR → Outcome)	−0.13	0.11	**−0.54, -0.01**	10
^c^Indirect effect (Aspirin → TWA_RDW → Outcome)	−0.09	0.12	−0.46, 0.01	6.9
^d^Indirect effect (Aspirin → TWA_PF → Outcome)	0.07	0.09	−0.21, 0.18	5.4
^e^Indirect effect (Aspirin → TWA_Cdyn → Outcome)	−0.24	0.16	−0.50, 0.17	18.5
^f^Indirect effect (Aspirin → TWA_WMP → Outcome)	−0.10	0.14	−0.57, 0.02	7.7
Total indirect effect (b + c + d + e + f)	−0.49	0.31	**−1.20, -0.07**	37.7

TWA, time weighted average; NLR, neutrophil to lymphocyte ratio; RDW, red cell distribution width; PF, oxygenation index; Cdyn, pulmonary dynamic compliance; WMP, mechanical power normalized to predicted body weight. Bold font indicate a significant statistical difference by multiple mediation analysis. a, b, c, d, e, and f represent the effect size in different mediation path.

### 3.6 Multiple mediation effect analysis

**FIGURE 3:**
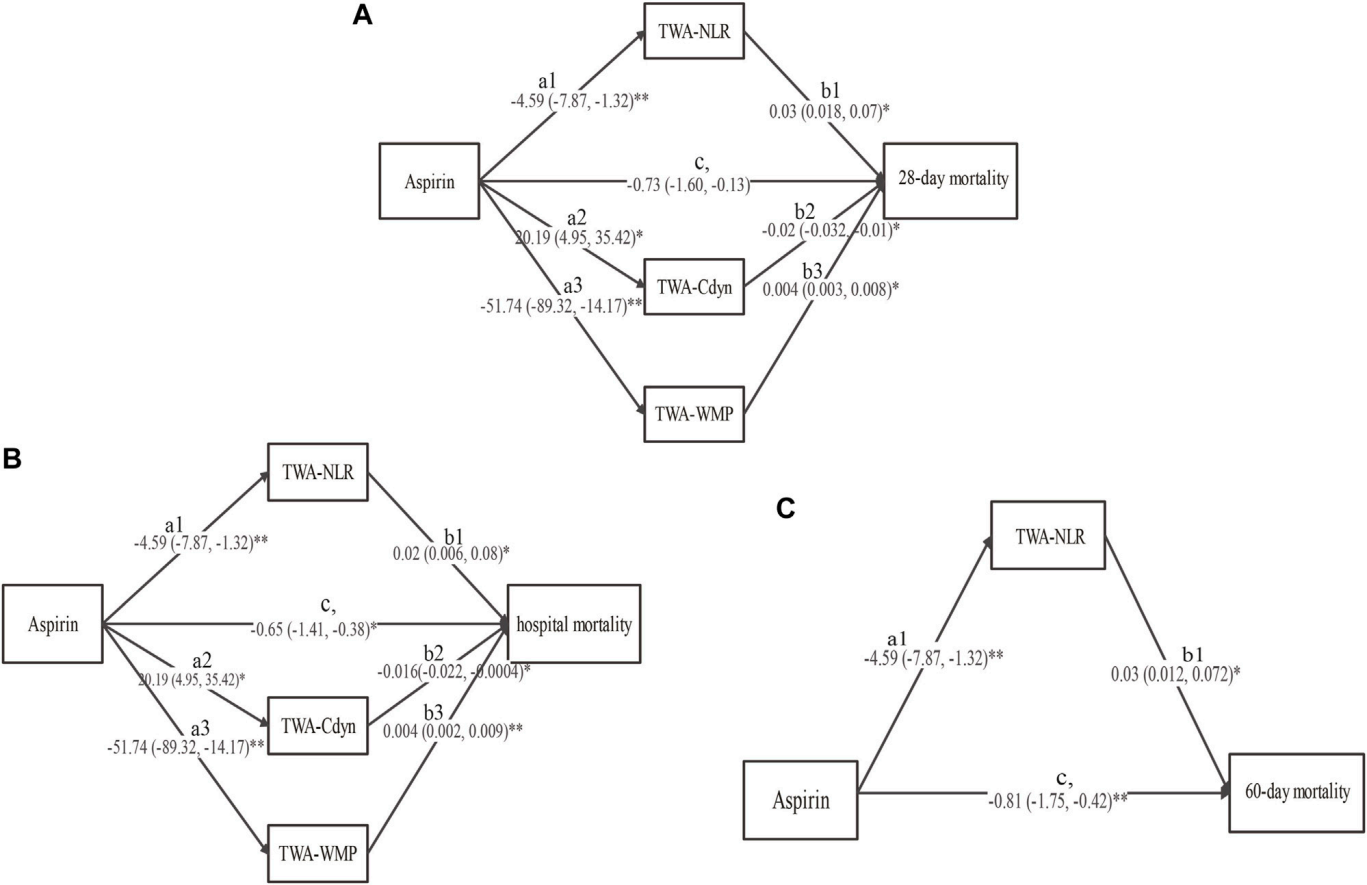
The results of the multiple mediation analysis between low-dose aspirin exposure before ICU admission and **(A)** 28-day mortality, **(B)** hospital mortality, and **(C)** 60-day mortality. Solid line indicates a significant effect in the path, *p < 0.05, **p < 0.01. TWA-RDW: time-weighted average red cell distribution width, TWA-NLR: time-weighted average neutrophil to lymphocyte ratio, TWA-Cdyn: time-weighted average dynamic lung compliance, TWA-WMP: time-weighted average mechanical power normalized to predicted body weight (TWA-WMP).

The comparisons for the TWA of each variable were presented in Supplementary Figure S14. The analysis revealed that low-dose aspirin exposure was negatively related to TWA-NLR (a1, -4.59, 95%CI, −7.87 to −1.32, *p* = 0.006) and TWA-WMP (a3, -51.74, 95%CI, −89.32 to −14.17, *p* = 0.007), and was positively related to TWA-Cdyn (a2, 20.19, 95%CI, 4.95 to 35.42, *p* = 0.01). For the 28-day mortality, the proposed mediators were TWA-NLR, TWA-Cdyn, and TWA-WMP. TWA-NLR (b1, 0.03, 95%CI, 0.018 to 0.07, *p* = 0.038) and TWA-WMP (b3, 0.004, 95%CI, 0.003 to 0.008, *p* = 0.033) were positively related to 28-day mortality, while the TWA-Cdyn (b2, -0.02, 95%CI, −0.032 to −0.01, *p* = 0.041) was negatively related to the 28-day mortality. Low-dose aspirin exposure was negatively related to the 28-day mortality (c^,^, −0.73, 95%CI, −1.60 to −0.13, *p* = 0.01) (Figure 3A and Supplementary Table S7).

For hospital mortality, the proposed mediators were TWA-NLR, TWA-Cdyn, and TWA-WMP. TWA-NLR (b1, 0.02, 95%CI, 0.006 to 0.08, *p* = 0.043) and TWA-WMP (b3, 0.004, 95%CI, 0.002 to 0.009, *p* = 0.009) were positively related to the hospital mortality, while the TWA-Cdyn (b2, -0.016, 95%CI, −0.022 to −0.0004, *p* = 0.047) was negatively related to the hospital mortality. Low-dose aspirin exposure was negatively related to hospital mortality (c^,^, −0.65, 95%CI, −1.41 to −0.38, *p* = 0.026) (Figure 3B and Supplementary Table S7).

For the 60-day mortality, the proposed mediator was only the TWA-NLR. TWA-NLR was positively related to the 60-day mortality (b1, 0.03, 95%CI, 0.012 to 0.072, *p* = 0.044). Low-dose aspirin exposure was also negatively related to the 60-day mortality (c^,^ *,* −0.81, 95%CI, −1.75 to −0.42, *p* = 0.003) (Figure 3C and Supplementary Table S7).

### 3.7 Bootstrap test of mediators

The results of the bootstrap estimation procedure with 10,000 bootstrap samples were presented in Table 3. Low-dose aspirin exposure was found to indirectly affect 28-day mortality through three significant mediation pathways: 1) TWA-NLR (effect, −0.14, 95%CI, −0.55 to −0.04), which accounted for 8.6% of the total effect; 2) TWA-Cdyn (effect, −0.40, 95%CI, −0.67 to −0.03), which accounted for 24.7% of the total effect; 3) TWA-WMP (effect, −0.21, 95%CI, −0.54 to −0.10), which accounted for 13% of the total effect. The total mediating effect was 54.9%.

Low-dose aspirin exposure was found to indirectly affect hospital mortality through three significant mediation pathways: 1) TWA-NLR (effect, −0.09, 95%CI, −0.64 to −0.04), which accounted for 6.5% of the total effect; 2) TWA-Cdyn (effect, −0.32, 95%CI, −0.74 to −0.01), which accounted for 23% of the total effect; 3) TWA-WMP (effect, −0.21, 95%CI, −0.65 to −0.04), which accounted for 15.1% of the total effect. The total mediating effect was 53.2%.

Low-dose aspirin exposure was found to indirectly affect 60-day mortality through one significant mediation pathway: TWA-NLR (effect, −0.13, 95%CI, −0.54 to −0.01), which accounted for 10% of the total effect. The total mediating effect was 37.7%.

## 4 Discussion

Pre-ICU aspirin exposure conferred a protective effect for patients with S-ARF that was mainly present in lower 28-day mortality, 60-day mortality, and hospital mortality. However, the findings of subgroup analysis showed that this protective effect given by aspirin exposure before ICU admission disappeared in those who did not receive IMV treatment, combined with acute myocardial infarction, or took high-dose aspirin. This phenomenon was also consistent with our further sensitivity analysis. Subsequently, in the beneficiary population, we analyzed the longitudinal values of NLR, RDW within the first 5 days after ICU admission and the trend of Cdyn, P/F, MP, and WMP within the 72 h after initial IMV between low-dose aspirin exposure group and non-aspirin exposure group. And the results of GAMM demonstrated that the initial levels of NLR, RDW, MP, and WMP in the low-dose aspirin exposure group were significantly lower than that in the non-aspirin exposure group; further, the Cdyn and P/F improved pronouncedly in the low-dose aspirin exposure group after IMV. Subsequent multiple mediation effect analysis also revealed that TWA-NLR, TWA-RDW, and TWA-WMP mediated the relationship between pre-ICU aspirin exposure and lower mortality. The additional risk of major gastrointestinal hemorrhage should be noted in the elderly and those combined with liver disease or thrombocytopenia during ICU admission, despite no significant difference in the overall population.

Our findings were generally consistent with the results of previously published studies (Boyle et al., 2015; Hsu et al., 2022; Lavie et al., 2022), although some differences exist, such as aspirin dosage, duration, and included population. Despite no guidelines for determining the optimal aspirin dose in sepsis or its related pulmonary complications therapy, it may be possible to get some inspiration based on pharmacological mechanisms (Eisen, 2012). For low doses, aspirin irreversibly acetylates COX-1, blocking the formation of thromboxane A2 and inhibiting platelet aggregation. By contrast, high-dose aspirin could downregulate the NF-κB pathway by selective inhibition of COX-2. In addition, the low-dose aspirin could generate an anti-inflammatory effect mediated by acetyl-salicylic acid-triggered 15-epi-lipoxin A4, and inhibited polymorphonuclear leukocyte (PMN)-platelet interactions that subsequently slowed the directed migration of PMN to the site of inflammation and strengthen the ability to modulate the immune response (Morris et al., 2009). Given its effectiveness and less toxicity, low-dose aspirin has been proposed as a potential drug for septic patients. Evidence from the Lavie et al. suggested that 75–100 mg/d aspirin for at least 30 days before hospitalization appeared to be effective in reducing 30-day and 90-day mortality in septic patients hospitalized in internal medicine wards (Lavie et al., 2022). However, another recent RCT reached the opposite conclusion indicating that 100 mg/d aspirin exposure was not associated with a decreased sepsis-related death risk for older patients (Eisen et al., 2021). This controversy might be caused by differences in population selection and the duration of therapy, while the latter may be more significant. A previous study indicated that the half maximal inhibitory concentration of acetylsalicylic acid for NF-κB inhibition is 5.67 mM. Despite the aspirin pharmacokinetic results in critically ill patients still unknown, an *in vivo* study based on health population has proved that the peak plasma concentration and thromboxane B2 inhibition were not increased substantially following 7 days of 80 mg aspirin daily (Cerletti et al., 2003). By sensitivity analysis, we also found that aspirin use during ICU stays would not influence the overall results (Supplementary Figure S15). These evidences demonstrated that low-dose aspirin exposure appears to be associated with improved outcomes in a duration-dependent manner. It also explains the negative results in a recent placebo-controlled RCT (STAR) (Toner et al., 2022). In this study, the majority of the aspirin dose used in the two groups were 81 mg/d and 325 mg/d, respectively. The results suggested that high-dose aspirin exposure prior to ICU admission did not significantly impact the outcomes for S-ARF patients, which is consistent with the previous study (Lavie et al., 2022).

Chronic low-dose aspirin is conventionally used to prevent cardiovascular/cerebrovascular disease and is not yet routinely recommended for patients with sepsis or ARF at present. Thus, we adjusted coronary or cerebrovascular comorbidities as confounders in AIPW rather than upfront exclude. Our subsequent subgroup analysis let to the discovery that pre-ICU aspirin exposure cannot efficiently reduce mortality in S-ARF patients admitted to CCU, indicating that aspirin protection is insufficient to counter the damage brought by acute CHD episodes. Interestingly, we also found a remarkably different between IMV and non-IMV group. Pre-ICU aspirin exposure showed a notable improvement in outcomes for patients who received IMV within ICU stay rather than those who did not. We speculate that this phenomenon may be related to the degree of acute lung injury (ALI). ARF and ARDS could be seen as a presentation of ALI under severe infections, while the latter is often accompanied by severe respiratory failure requiring IMV curing. Therefore, we believe there is a significant need to examine the similarity between the IMV and ARDS subgroups and assess pre-ICU aspirin’s effect on mortality in patients diagnosed with ARDS after ICU admission. Berlin criteria and the International Classification of Diseases were commonly used for ARDS patient screening in the MIMIC-IV database. However, both methods remain some limitations. For example, we cannot obtain accurate chest radiography time, and no ARDS-specific codes exist in ICD-9. In this study, we extracted the imaging characteristics of ARDS from chest radiograph reports using natural language processing in MIMIC-CXR and then matched the report time with other features as far as possible. Finally, a total of 181 patients met the Berlin criteria, 180 of whom received the IMV, and only 18 of whom had taken aspirin before ICU admission. Thus, we were unable to perform further analysis in the ARDS subgroup.

Another important discovery of this study is that low-dose aspirin exposure before ICU admission alleviated the early inflammatory status of S-ARF patients and facilitated the improvement of P/F and Cdyn after IMV initialing. As an indicator of systemic inflammation, NLR and RDW have been frequently used to predict outcomes for sepsis and ARDS patients in the previous studies (Yoo et al., 2020; Liu et al., 2021; Wang et al., 2021). Nevertheless, most of them only used the initial value on admission or a single measurement at a particular moment. It would not reflect the dynamic change of the inflammatory response and its effects on prognosis dynamics. Based on the MIMIC-IV database, a study published in 2021 illustrated a significant difference in the NLR trend within 7 days after ICU admission between the survival and death group for ARDS patients (Zhang et al., 2021). Regrettably, the dynamic change of NLR or RDW of the populations with a low-dose aspirin medication after suffering infections has so far not been investigated. This present study found significantly lower initial NLR (*β*, −3.83, SE, 1.43, *p* = 0.008) and RDW (*β*, −0.60, SE, 0.27, *p* = 0.027) levels in S-ARF patients who took low-dose aspirin compared with those not taking it. Despite no differences in slope, the TWA-NLR (11.06 [IQR, 7 to 16.62] vs. 7.43 [IQR, 4.52 to 12.1], *p* < 0.001) in the low-dose aspirin group is significantly lower than that in the non-aspirin group within the 5 day after ICU admission. This result is similar to ARENA’s conclusion evaluating the effect of aspirin on pulmonary inflammation in ARDS patients induced by lipopolysaccharide (LPS) (Hamid et al., 2017). The ARENA trial carried in Northern Ireland randomized 33 health volunteers to three groups. Patients in the two treatment groups received 75 mg/d and 1200 mg/d aspirin for 7 days before LPS inhalation, while those in the control group only took a placebo. They finally found that aspirin could reduce the neutrophilia counts in bronchoalveolar lavage fluid at the 6th hour after LPS inhalation. However, we should note that the lung injury under the testing LPS concentration (50 μg) was limited; further, this model did not sufficiently represent the complex pathological manifestations when suffering polymicrobial infections.

Both P/F and Cdyn are important features reflecting lung inflammation reduction and respiratory function improvement, closely related to clinical outcomes. We demonstrated that when comparing with the non-aspirin group, the Cdyn and P/F ratio increased by 0.31 mL/cmH_2_O and 0.43 mmHg every hour in the low-dose aspirin exposure group within the first 72 h after receiving IMV initialing. This phenomenon can be well explained by a resolved pulmonary edema and microthrombi. Besides, by the multiple mediation analysis, we also revealed that TWA-Cdyn during the 72 h after IMV was an essential mediator for the protective effect of low-dose aspirin in the 28-day mortality and hospital mortality. MP presents the summary value for the energy transmitted from the ventilator to the respiratory system per unit of time, while WMP is the absolute MP value when normalized to predicted body weight, which balances the discrepancy induced by the functional lung size (Tonetti et al., 2017; Zhang et al., 2019). Previous clinical studies suggested that an increased MP and WMP might produce ventilator-induced lung injury, although IMV therapy is a mainstay of ARF and ARDS management (Zhang et al., 2019; Urner et al., 2020). Interestingly, in this study, the initial MP and WMP values of patients in the low-dose aspirin group were significantly lower than that in the non-aspirin group within the first 12 h which provided a new mechanism for the potential protective effect of low-dose aspirin.

### 4.1 Limitations

The limitations of this study need to be acknowledged. The first limitation came from other potential confounders. Although we minimized the structural confounding bias using DGA and generated a doubly robust unbiased estimation through the AIPW approach, it is impossible to adjust for all confounders, including other medications or the administration condition about aspirin during ICU stay, for an observational study. Thus, the conclusions should be interpreted with caution. The difficulty in obtaining the medication history and adherence was the second limitation of this study. We have excluded those who took aspirin until after ICU admission to screen out the potential target population taking aspirin chronically wherever possible. However, as a public database rather than a purposely designed cohort, we could not trace the treatment duration and medication adherence. Follow-up studies should elucidate the correlations between the duration of aspirin exposure and treatment effects in different populations. In addition, the degree of emphasis on health states is another important consideration. The patients with chronic aspirin medicine tended to assign greater importance to their self-health states. Lastly, only 4% of included S-ARF patients did not receive MV treatment. Thus, the subsequent subgroup analysis was just stratified by whether the individual had a history of IMV curing. However, given the differences across facilities, a better understanding of the effect of pre-ICU aspirin exposure on those receiving therapies of differing ventilatory supports is needed.

## 5 Conclusion

In summary, pre-ICU aspirin exposure was associated with decreased mortality in adult patients with S-ARF and did not increase the additional thrombocytopenia and major gastrointestinal hemorrhage during ICU stay. The protective effect of low-dose aspirin exposure before ICU admission may be mediated by a low dynamic level of NLR, WMP, and a high dynamic level of Cdyn. The results of this single-center study need to be validated at the multicenter level.

## Data Availability

The original contributions presented in the study are included in the article/Supplementary Material, further inquiries can be directed to the corresponding authors.

## Abbreviations

S- ARF, Sepsis-associated acute respiratory failure; ICU, intensive care unit; ATE, average treatment effect; NLR, neutrophil to lymphocyte ratio; RDW, red cell distribution width; P/F, oxygenation index; Cdyn, dynamic lung compliance; MP, mechanical power; WMP, mechanical power normalized to predicted body weight; IMV, invasive mechanical ventilation; COX, cyclooxygenase; NF-κB, nuclear factor kappa B; ARDS, acute respiratory distress syndrome; AIPW, augmented inverse probability weighting; GAMM, generalized additive mixed model; SOFA, Sequential Organ Failure Assessment; SAPS II, Simplify Acute Physiological Scores II; MFI, myocardial infarction; CIF, cerebral infarction; CKD, chronic kidney disease; HF, heart failure; LD, liver disease; ACEI/ARB, angiotensin-converting enzyme inhibitors, and angiotensin receptor blockers; PSM, propensity score matching; TWA, time-weighted average; ALI, acute lung injury.
