# Supplementary material for: The impact of aspirin exposure prior to intensive care unit admission on the outcomes for patients with sepsis-associated acute respiratory failure
Source: Front Pharmacol. 2023 Mar 1;14:1125611. doi: 10.3389/fphar.2023.1125611 (PMC10014538; doi:10.3389/fphar.2023.1125611)
Supplement: Supplementary file 1 [file DataSheet1.DOCX]

**The Impact of Aspirin Exposure Prior to Intensive Care Unit Admission on the Outcomes for Patients with Sepsis-Associated Acute Respiratory Failure**

**Supplementary Materials**

Table S1: The ICD-9 and ICD-10 codes used to screen for comorbidities.

| **Comorbidities** | **ICD-9 codes** | **ICD-10 codes** |
| --- | --- | --- |
| Diabetes mellitus (DM) | 25000-25003, 25010-25013, 25020-25023, 25030-25033, 25040, 24900, 24901, 24910, 24911, 24920, 24921, 24930, 24931, 24940, 24941, 24950, 24951, 24960, 24961, 24970, 24971, 24980, 24981, 24990, 24991, 25041-25043, 25050-25053, 25060-25063, 25070-25073, 25080-25083, 25090-25093, 5881 | E100, E10l, E106, E108, E109,E110,E111, E116,E118, E119,E120,E121,E126,E128,E129,E130,E131,E136,E138,E139,  E140,E141,E146,E148,E149 |
| Hypertension (HT) | 4011, 4019 | I10, I15, I518, I519 |
| Coronary heart disease (CHD) | 41403, 41400-41402 ,41404-41407 ,4143 ,4144 | I2583, I2584 |
| Acute or chronic heart failure (HF) | 39891, 40201, 40211, 40291, 40401, 40403, 40411, 40413, 40491, 40493, 4280, 4281, 42820-42823, 42830-42833, 42840-42843, 4289 | I0981,I110,I119,I130,I131,I1310,I1311,I132,I502,I5020,I5021,I5022,I5023,I503,I5030,I5031, I5032,I5033,I504,I5040,I5041,I5042,I5043,I508,I5081,I50810,I50811,I50812,I50813,I50814, I5082,I5083,I5084,I5089,I9713,I97130,I97131 |
| [Cerebral](javascript:;) [infarction](javascript:;) | 34660,34661,34662,34663,43301,43311,43321,43331,43381,43391,43401,43411,43491 | G436,G4360,G43601,G43609,G4361,G43611,G43619,I63,I630,I6300,I6301  ,I63011,I63012,I63013,I63019,I6302,I6303,I63031,I63032,I63033,I63039,I6309  ,I631,I6310,I6311,I63111,I63112,I63113,I63119,I6312,I6313,I63131,I63132,I63133  ,I63139,I6319,I632,I6320,I6321,I63211,I63212,I63213,I63219,I6322,I6323,I63231  ,I63232,I63233,I63239,I6329,I633,I6330,I6331,I63311,I63312,I63313,I63319,I6332  ,I63321,I63322,I63323,I63329,I6333,I63331,I63332,I63333,I63339,I6334,I63341,I63342  ,I63343,I63349,I6339,I634,I6340,I6341,I63411,I63412,I63413,I63419,I6342,I63421,I63422  ,I63423,I63429,I6343,I63431,I63432,I63433,I63439,I6344 ,I63441,I63442,I63443,I63449  ,I6349,I635,I6350,I6351,I63511,I63512,I63513,I63519,I6352,I63521,I63522 ,I63523,I63529  ,I6353,I63531,I63532,I63533,I63539,I6354,I63541,I63542,I63543,I63549,I6359,I636,I638  ,I6381,I6389,I639,I65,I66,I693,I6930,I6931,I69310,I69311,I69312,I69313,I69314,I69315  ,I69318,I69319,I6932,I69320,I69321,I69322,I69323,I69328,I6933,I69331,I69332,I69333  ,I69334,I69339,I6934,I69341,I69342,I69343,I69344,I69349,I6935,I69351,I69352,I69353  ,I69354,I69359,I6936,I69361,I69362,I69363,I69364,I69365,I69369,I6939,I69390,I69391  ,I69392,I69393,I69398,Z8673 |
| Myocardial infarction | 41000,41001,41002,41010,41011,41012,41020,41021,41022,41030,41031,41032,41040,41041,41042,41050,41051,41052,41080,41081,41082,41090,41091,41092,4110  ,41181,412 | I21,I210,I2101,I2102,I2109,I211,I2111,I2119,I212,I2121  ,I2129,I213,I214,I219,I21A,I21A1,I21A9,I22,I220,I221  ,I222,I228,I229,I252 |
| COPD | 490,496,500,501,502,4168,4169,4910,4911,4918,4919,4920,4928,4940,4941,4952,4957,4958,4959,5081,5088,49120,49121,49122,49300,49301,49302,49310,49312,49320,49321,49322  ,49381,49382,49390,49391,49392 | I2781,I2782,I2783,I279,J40,J410,J411,J42,J430,J431,J432,J438,J439,J440,J441,J449 ,J4520,J4521,J4522,J4530,J4531,J4540,J4541,J4550,J4551,J4552,J45901,J45902,J45909,J45990,J45991,J45998,J470,J471,J479 ,J60,J61,J628,J632,J634,J636,J64,J662,J668,J672,J678,J679,J684,J701,J703 |
| Chronic kidney disease (CKD) | 40300, 40301, 40310, 40311 , 40390, 40391, 40400-40403, 40410-40413 , 40490-40493, 5851-5855, 5859 | E0822 ,E0922,E1022,E1122,E1322,I12,I120,I129,I13,I130,I131,I1310  ,I1311,I132,N18,N181,N182,N183,N184,N185,N189 |
| Upper gastrointestinal bleeding | 4560,45620,53021 | I8501,I8511,K2211,K2901,K2961,2971,K2981,K2991,K31811 |

Table S2: The ICD-9 and ICD-10 codes used to screen infection classification.

| **Infection classification** | **ICD-9** | **ICD-10** |
| --- | --- | --- |
| Pulmonary infections | 46430,48284,48239,4843,51189,48230,4871,486,46450,5118,4801,  481,8289,4838,4829,4809,46611,48283,46400,4821,4660,4808,5111,  5110,462,4650,48231,4611,46421,4830,4659,48241,463,4658,485,  4802,4848,4618,4613,4820,4800,4846,5119,48249,46411,46619,  48282,48232,4847,4822,48242,4841,4610,4870,5100,48281,4619,  46451,460,46410,4878,46431,5109,51181,48240,4612 | J151,J09X3,J09X1,J1289,J101,J156,J211,J0190,J0180,J0390,  J0410,J0511,J09X9,J0141,J219,J205,J111,J1100,J120,J102,  J129,J1529,J189,J153,J0120,J00,J123,J201,J154,J0191,J210,  J209,J020,J155,J0411,J188,J17,J13,J122,J042,J158,J181,J15211,  J218,J112,J0300,J1001,J1520,J851,J1008,J157,J1000,J208,J852,  J22,J0380,J14,J869,J0130,J0391,J1089,J0140,J853,J040,J0431,  J150,J204,J168,J182,J1081,J09X2,J180,J0430,J0301,J1108,J028,  J0100,J860,J121,J0510,J850,J0110,J069,J029,J060,J1082,J1189,  J1181,J159,J15212 |
| Urinary tract infections | 0980,0981,0982,0983,5990 | A540,A5400,A5409,A541,A560,A5600,A5609,A562,N390, N99521,N99531,N390 |
| Skin subcutaneoustissue infections | 680-686, 690-698 | L00-L08 |
| Catheter related infections | 99664, 99668,99931, 99932, 99933 | T8021, T8351, T8571,T8571,T85735 |
| Intestinal infections | 00867,0069,0074,00589,00846,0059,0048,00809,00804,0041,  00843,0088,0062,0040,0043,0093,0020,0078,0091,00841,0071,  0049,0090,0030,00800,00844,0083,00324,00845,0050,0085,  00863,0039,00581,00861,00849,0031,00869,00329,0038,0068,  0029,00847,0092,00862 | A048,A039,A088,A021,A0811,A030,A0471,A046,A0100,A059,  A0839,A082,A029,A033,A028,A078,A047,A069,A071,A080,  A072,A0222,A0472,A050,A084,A09,A043,A049,A038,A031,  A044,A045,A020 |
| Septicemia | 038 | A41, A40 |

Table S3: Baseline characteristics of sepsis associated acute respiratory failure extracted from MIMIC-IV (2.0) database.

| **Variables** | **Total (n = 2090)** | **No aspirin exposure (n = 1469)** | **Pre-ICU aspirin exposure (n = 621)** | **p** |
| --- | --- | --- | --- | --- |
| **Demographic features** |  |  |  |  |
| Age, (year) | 65.44 (53.29, 77.32) | 60.89 (49.66, 73.61) | 73.44 (64.41, 80.57) | < 0.001 |
| Gender, n (%) |  |  |  | 0.008 |
| Female | 919 (44) | 674 (46) | 245 (39) |  |
| Male | 1171 (56) | 795 (54) | 376 (61) |  |
| Race, n (%) |  |  |  | < 0.001 |
| ASIAN | 42 (2) | 34 (2) | 8 (1) |  |
| BLACK | 186 (9) | 138 (9) | 48 (8) |  |
| HISPANIC/LATINO | 69 (3) | 48 (3) | 21 (3) |  |
| WHITE | 1306 (62) | 869 (59) | 437 (70) |  |
| OTHER | 84 (4) | 68 (5) | 16 (3) |  |
| UNKNOWN | 403 (19) | 312 (21) | 91 (15) |  |
| Weight, (Kg) | 80 (66.8, 96.57) | 79.6 (65.8, 96.9) | 81.2 (68.4, 95.3) | 0.289 |
| Height, (cm) | 169.69 (165, 175) | 169.69 (165, 173) | 169.72 (163, 178) | 0.732 |
| BMI | 28.23 (24.33, 32.24) | 28.2 (24.2, 32.24) | 28.37 (24.77, 32.25) | 0.682 |
| **ICU type (%)** |  |  |  | < 0.001 |
| CCU | 316 (15) | 176 (12) | 140 (23) |  |
| CVICU | 370 (18) | 50 (3) | 320 (52) |  |
| MICU | 1389 (66) | 1230 (84) | 159 (26) |  |
| OTHER | 15 (1) | 13 (1) | 2 (0) |  |
| **Previous Medication (%)** |  |  |  |  |
| Statin | 469 (22) | 60 (4) | 409 (66) | < 0.001 |
| ACEI/ARB | 161 (8) | 21 (1) | 140 (23) | < 0.001 |
| **Comorbidity (%)** |  |  |  |  |
| COPD | 660 (32) | 427 (29) | 233 (38) | < 0.001 |
| Cerebral infarction | 137 (7) | 77 (5) | 60 (10) | < 0.001 |
| Myocardial infarction | 278 (13) | 109 (7) | 169 (27) | < 0.001 |
| Hypertension | 763 (37) | 499 (34) | 264 (43) | < 0.001 |
| Coronary heart disease | 384 (18) | 75 (5) | 309 (50) | < 0.001 |
| Heart failure | 718 (34) | 365 (25) | 353 (57) | < 0.001 |
| Diabetes | 637 (30) | 365 (25) | 272 (44) | < 0.001 |
| Chronic kidney disease | 479 (23) | 266 (18) | 213 (34) | < 0.001 |
| Liver disease | 576 (28) | 514 (35) | 62 (10) | < 0.001 |
| **Infection sites (%)** |  |  |  |  |
| Pulmonary infections | 762 (36) | 580 (39) | 182 (29) | < 0.001 |
| Skin-subcutaneoustiss infections | 180 (9) | 129 (9) | 51 (8) | 0.735 |
| Catheter related infections | 51 (2) | 36 (2) | 15 (2) | 1 |
| Urinary tract infections | 382 (18) | 270 (18) | 112 (18) | 0.901 |
| Intestinal infections | 121 (6) | 89 (6) | 32 (5) | 0.479 |
| Septicemia | 763 (37) | 653 (44) | 110 (18) | < 0.001 |
| **Vital signs** |  |  |  |  |
| Mean heart rate, (min^−1^) | 87.86 (77.47, 100.77) | 90.77 (78.17, 104.15) | 83.92 (75.92, 93.03) | < 0.001 |
| Mean MBP, (mmHg) | 74.36 (69.05, 80.1) | 74.93 (69.3, 80.88) | 73.54 (68.7, 78.96) | < 0.001 |
| Mean SBP, (mmHg) | 109.58 (102.72, 118.32) | 109.31 (102.33, 118.5) | 110.54 (103.91, 117.9) | 0.348 |
| Mean DBP, (mmHg) | 60.48 (54.44, 66.87) | 61.77 (55.88, 68.19) | 57 (51.87, 63.48) | < 0.001 |
| Mean respiratory rate, (min^−1^) | 20.01 (17.41, 23.38) | 20.62 (17.81, 24.16) | 18.74 (16.68, 21.81) | < 0.001 |
| Mean temperature, (℃) | 36.88 (36.55, 37.21) | 36.89 (36.57, 37.29) | 36.87 (36.51, 36.99) | < 0.001 |
| Urineoutput, (ml) | 1350 (730, 2176.5) | 1306 (680, 2160) | 1430 (870, 2200) | 0.052 |
| **Laboratory tests^a^** |  |  |  |  |
| Minimum arterial lactate, (mmol/L) | 1.51 (1.2, 2.2) | 1.55 (1.2, 2.42) | 1.3 (1, 1.7) | < 0.001 |
| Maximum arterial lactate, (mmol/L) | 2.99 (1.7, 4.2) | 2.99 (1.7, 4.16) | 3 (1.8, 4.3) | 0.046 |
| Minimum arterial PH | 7.3 (7.23, 7.36) | 7.3 (7.23, 7.37) | 7.3 (7.24, 7.35) | 0.812 |
| Maximum arterial PH | 7.42 (7.37, 7.46) | 7.41 (7.36, 7.45) | 7.44 (7.4, 7.48) | < 0.001 |
| Minimum PaO2, (mmHg) | 72 (54.25, 85) | 70 (52, 84.06) | 76 (60, 94) | < 0.001 |
| Maximum PaO2, (mmHg) | 173 (102.25, 293.75) | 155 (96, 237.76) | 336 (146, 425) | < 0.001 |
| Minimum PaCO2, (mmHg) | 35.99 (30, 39) | 36.08 (31, 40) | 34 (30, 38) | < 0.001 |
| Maximum PaCO2, (mmHg) | 49 (40, 55) | 48.83 (39, 54) | 49 (43, 56) | < 0.001 |
| Minimum base excess, (mmol/L) | -3.53 (-7, 0) | -3.53 (-7, 0) | -3.62 (-7, -1) | 0.423 |
| Maximum base excess, (mmol/L) | 0 (-2, 2) | 0 (-3, 1.15) | 1.02 (0, 4) | < 0.001 |
| Minimum bicarbonate, (mmol/L) | 20 (17, 23) | 19 (16, 23) | 22 (19, 24) | < 0.001 |
| Maximum bicarbonate, (mmol/L) | 24 (21, 27) | 23 (20, 26) | 25 (22, 27) | < 0.001 |
| Minimum anion gap, (mEq/L) | 13 (11, 16) | 14 (11, 16) | 12 (10, 15) | < 0.001 |
| Maximum anion gap, (mEq/L) | 17 (14, 21) | 18 (15, 22) | 16 (13, 19) | < 0.001 |
| Minimum RDW, (%) | 15 (13.9, 16.8) | 15.2 (14, 17.2) | 14.6 (13.7, 15.8) | < 0.001 |
| Maximum RDW, (%) | 15.4 (14.2, 17.3) | 15.6 (14.2, 17.7) | 15.1 (14.2, 16.4) | < 0.001 |
| Minimum platelets, (×10^9/L) | 150 (95.25, 223) | 148 (85, 226) | 154 (110, 218) | 0.019 |
| Maximum platelets, (×10^9/L) | 198.5 (134, 277) | 194 (119, 279) | 209 (155, 274) | < 0.001 |
| Minimum hemoglobin, (g/dL) | 9.3 (7.9, 11) | 9.6 (8, 11.3) | 8.9 (7.7, 10.1) | < 0.001 |
| Maximum hemoglobin, (g/dL) | 11 (9.6, 12.7) | 11 (9.4, 12.8) | 11.1 (9.9, 12.5) | 0.172 |
| Minimum hematocrit, (vol%) | 28.3 (24, 33.68) | 29.2 (24.7, 34.8) | 26.4 (23.3, 30.8) | < 0.001 |
| Maximum hematocrit, (vol%) | 33.6 (29.4, 38.7) | 33.7 (28.9, 39.3) | 33.4 (30.2, 37.4) | 0.988 |
| Minimum glucose, (mg/dl) | 109 (90, 135) | 107 (88, 133) | 116 (97, 139) | < 0.001 |
| Maximum glucose, (mg/dl) | 154 (121, 210) | 155 (123, 209) | 148 (116, 214) | 0.027 |
| Minimum WBC, Median (K/uL) | 10.2 (7.1, 13.9) | 10.5 (7, 14.2) | 9.8 (7.2, 13) | 0.105 |
| Maximum WBC, Median (K/uL) | 14.8 (10.6, 20.1) | 14.6 (10.3, 20.3) | 15.1 (11.4, 19.7) | 0.375 |
| Minimum lymphocytes, (K/uL) | 1.09 (0.67, 1.36) | 1.03 (0.57, 1.34) | 1.33 (1.05, 1.38) | < 0.001 |
| Maximum lymphocytes, (K/uL) | 1.31 (0.83, 1.6) | 1.23 (0.73, 1.58) | 1.55 (1.2, 1.61) | < 0.001 |
| Minimum neutrophils, (K/uL) | 10.12 (7.59, 12.84) | 10.13 (6.9, 13.13) | 10.11 (9.75, 12.48) | 0.169 |
| Maximum neutrophils, (K/uL) | 11.45 (8.63, 14.61) | 11.46 (7.85, 15.21) | 11.44 (10.77, 14.02) | 0.303 |
| Minimum BUN, (mg/dL) | 23 (14, 41) | 25 (14, 43) | 21 (14, 37) | 0.015 |
| Maximum BUN, (mg/dL) | 29 (18, 51) | 31 (19, 54) | 26 (18, 44) | < 0.001 |
| Minimum creatinine, (μmol/L) | 1.1 (0.7, 1.8) | 1.1 (0.7, 1.9) | 1.1 (0.8, 1.8) | 0.951 |
| Maximum creatinine, (μmol/L) | 1.4 (0.9, 2.4) | 1.5 (0.9, 2.5) | 1.3 (0.9, 2.2) | 0.169 |
| Minimum bilirubin (total), (μmol/L) | 1.1 (0.5, 1.7) | 1 (0.4, 2.2) | 1.49 (0.6, 1.59) | 0.462 |
| Maximum bilirubin (total), (μmol/L) | 1.5 (0.52, 2.1) | 1.3 (0.5, 2.9) | 1.75 (0.7, 1.85) | 0.621 |
| Minimum blood sodium, (mEq/L) | 137 (133.25, 140) | 137 (133, 140) | 137 (134, 139) | 0.282 |
| Maximum blood sodium, (mEq/L) | 140 (137, 143) | 140 (137, 144) | 140 (137, 142) | 0.023 |
| Minimum blood potassium, (mEq/L) | 3.9 (3.4, 4.3) | 3.8 (3.4, 4.3) | 4 (3.6, 4.4) | < 0.001 |
| Maximum blood potassium, (mEq/L) | 4.6 (4.1, 5.2) | 4.6 (4.1, 5.3) | 4.7 (4.3, 5.1) | 0.194 |
| **Severity scoring** |  |  |  |  |
| SOFA | 4 (3, 5) | 4 (3, 6) | 4 (3, 5) | 0.185 |
| SAPSII | 43 (35, 53) | 43 (34, 53) | 43 (35, 53) | 0.534 |
| Charlson comorbidity index | 6 (4, 8) | 5 (3, 7) | 7 (5, 9) | < 0.001 |
| GCS scores | 12 (6.25, 14) | 11 (6, 14) | 13 (8, 14) | < 0.001 |
| **Treatments during ICU stay** |  |  |  |  |
| Vasoactive drug | 1288 (62) | 852 (58) | 436 (70) | < 0.001 |
| Mechanical ventilation | 2004 (96) | 1395 (95) | 609 (98) | 0.002 |
| CRRT | 306 (15) | 222 (15) | 84 (14) | 0.385 |
| **Outcomes** |  |  |  |  |
| 28-day mortality, n (%) | 606 (29) | 479 (33) | 127 (20) | < 0.001 |
| 60-day mortality, n (%) | 696 (33) | 539 (37) | 157 (25) | < 0.001 |
| Hospital mortality, n (%) | 538 (26) | 419 (29) | 119 (19) | < 0.001 |
| ICU length of stay, (day) | 5.05 (3.16, 8.9) | 5.08 (3.08, 8.92) | 4.98 (3.26, 8.77) | 0.834 |
| Thrombocytopenia, n(%) | 835 (40) | 615 (42) | 220 (35) | 0.007 |
| Major gastrointestinal hemorrhage, n (%) | 93 (4) | 59 (4) | 34 (5) | 0.173 |

Table S4: The results of sensitivity analysis by multiple logistic regression model and propensity score matching.

| **Outcomes** | **multiple regression^a^** | | | **PSM^a^** | | |
| --- | --- | --- | --- | --- | --- | --- |
|  | **aOR/coefficient** | **95%CI** | ***P*** | **OR/coefficient** | **95%CI** | ***P*** |
| ICU 28-day mortality | 0.51 | 0.36-0.71 | **<0.001** | 0.49 | 0.32-0.73 | **<0.001** |
| ICU 60-day mortality | 0.56 | 0.40-0.78 | **<0.001** | 0.53 | 0.36-0.78 | **0.001** |
| Hospital mortality | 0.65 | 0.46-0.92 | **0.017** | 0.61 | 0.40-0.91 | **0.018** |
| ICU length of stay | 1.39 | 0.51-4.83 | 0.566 | 1.40 | 0.47-4.16 | 0.545 |
| Thrombocytopenia during ICU stay | 1.26 | 0.91-1.74 | 0.161 | 0.90 | 0.62-1.30 | 0.57 |
| Major bleeding risk | 1.35 | 0.70-1.86 | 0.108 | 1.14 | 0.59-2.34 | 0.633 |

a: adjusted for age, gender, BMI, SOFA, SAPSII, diabetes, cerebral infarction, myocardial infarction, hypertension, coronary heart disease, heart failure, chronic kidney disease, liver disease, statin, ACEI/ARB, pulmonary infection, septic shock.

Table S5: The results of sensitivity analysis stratified by received invasive mechanical ventilation or not and the initial aspirin dose.

| **Outcomes** | **Multiple regression^a^** | | | **PSM^a^** | | |
| --- | --- | --- | --- | --- | --- | --- |
|  | **aOR/coefficient** | **95%CI** | ***P*** | **OR/coefficient** | **95%CI** | ***P*** |
| **Receive invasive MV curing** |  |  |  |  |  |  |
| 28-day mortality | 0.35 | 0.22-0.53 | **<0.001** | 0.36 | 0.22-0.58 | **<0.001** |
| 60-day mortality | 0.42 | 0.28-0.62 | **<0.001** | 0.43 | 0.27-0.69 | **<0.001** |
| Hospital mortality | 0.48 | 0.32-0.73 | **<0.001** | 0.45 | 0.28-0.73 | **0.001** |
| ICU length of stay | 0.80 | 0.36-1.81 | 0.600 | 1.22 | 0.29-5.02 | 0.787 |
| Thrombocytopenia during ICU stay | 1.63 | 1.11-2.38 | **0.012** | 1.39 | 1.16-1.86 | **0.026** |
| Major bleeding risk | 1.58 | 0.94-4.69 | 0.201 | 1.75 | 0.76-5.21 | 0.295 |
| **No receive invasive MV curing** |  |  |  |  |  |  |
| 28-day mortality | 1.25 | 0.64-2.41 | 0.503 | 0.96 | 0.45-2.01 | 0.909 |
| 60-day mortality | 1.13 | 0.60-2.10 | 0.712 | 0.87 | 0.42-1.74 | 0.687 |
| Hospital mortality | 1.25 | 0.60-2.56 | 0.547 | 1.05 | 0.44-2.43 | 0.901 |
| ICU length of stay | 2.13 | 0.65-6.98 | 0.210 | 0.68 | 0.20-2.34 | 0.539 |
| Thrombocytopenia during ICU stay | 1.37 | 0.70-1.78 | 0.110 | 1.02 | 0.66-1.59 | 0.913 |
| Major bleeding risk | 1.16 | 0.76-3.58 | 0.144 | 2.56 | 0.97-8.03 | 0.075 |
| **Dose >100mg** |  |  |  |  |  |  |
| 28-day mortality | 0.97 | 0.48-1.96 | 0.938 | 0.36 | 0.98-1.18 | 0.107 |
| 60-day mortality | 0.96 | 0.48-1.89 | 0.902 | 0.53 | 0.16-1.58 | 0.267 |
| Hospital mortality | 1.08 | 0.52-2.22 | 0.826 | 0.53 | 0.14-1.78 | 0.321 |
| ICU length of stay | 3.18 | 0.46-21.72 | 0.238 | 3.22 | 0.42-24.52 | 0.257 |
| Thrombocytopenia during ICU stay | 1.32 | 0.48-2.14 | 0.454 | 0.81 | 0.31-1.96 | 0.643 |
| Major bleeding risk | 2.03 | 0.69-4.74 | 0.507 | 2.27 | 0.79-4.14 | 0.273 |
| **Dose ≤100mg** |  |  |  |  |  |  |
| 28-day mortality | 0.37 | 0.20-0.65 | **<0.001** | 0.35 | 0.16-0.73 | **<0.001** |
| 60-day mortality | 0.36 | 0.21-0.63 | **<0.001** | 0.44 | 0.21-0.88 | **0.024** |
| Hospital mortality | 0.45 | 0.24-0.81 | **0.009** | 0.42 | 0.18-0.92 | **0.035** |
| ICU length of stay | 2.20 | 0.52-9.30 | 0.284 | 1.91 | 0.37-5.83 | 0.438 |
| Thrombocytopenia during ICU stay | 1.48 | 0.86-2.55 | 0.154 | 1.11 | 0.63-1.93 | 0.721 |
| Major bleeding risk | 1.72 | 0.37-7.38 | 0.105 | 2.52 | 0.95-7.04 | 0.068 |
| **Admission in CCU** |  |  |  |  |  |  |
| 28-day mortality | 1.33 | 0.63-2.83 | 0.448 | 1.28 | 0.57-2.89 | 0.558 |
| 60-day mortality | 1.11 | 0.53-2.32 | 0.784 | 1.17 | 0.52-2.64 | 0.697 |
| Hospital mortality | 1.53 | 0.71-3.31 | 0.281 | 1.39 | 0.61-3.21 | 0.430 |
| ICU length of stay | 3.69 | 0.55-24.67 | 0.176 | 2.15 | 0.25-18.32 | 0.479 |
| Thrombocytopenia during ICU stay | 0.71 | 0.31-1.61 | 0.418 | 0.94 | 0.63-1.41 | 0.773 |
| Major bleeding risk | 2.09 | 0.33-6.42 | 0.521 | - | - | - |
| **Non CCU admission** |  |  |  |  |  |  |
| 28-day mortality | 0.41 | 0.27-0.62 | **<0.001** | 0.35 | 0.21-0.56 | **<0.001** |
| 60-day mortality | 0.51 | 0.35-0.75 | **<0.001** | 0.42 | 0.26-0.65 | **<0.001** |
| Hospital mortality | 0.55 | 0.36-0.83 | **0.005** | 0.45 | 0.27-0.73 | **0.002** |
| ICU length of stay | 2.18 | 0.79-6.01 | 0.133 | 1.25 | 0.36-4.40 | 0.725 |
| Thrombocytopenia during ICU stay | 1.39 | 0.97-1.99 | 0.073 | 0.94 | 0.63-1.41 | 0.773 |
| Major bleeding risk | 4.04 | 0.59-12.34 | 0.814 | 3.59 | 0.78-11.10 | 0.663 |

a: adjusted for age, gender, BMI, SOFA, SAPSII, diabetes, cerebral infarction, myocardial infarction, hypertension, coronary heart disease, heart failure, chronic kidney disease, liver disease, statin, ACEI/ARB, pulmonary infection, septic shock.

Table S6: Comparisons of red cell distribution width (RDW), neutrophil to lymphocyte ratio (NLR), PaO_2_/FiO_2_ (P/F), dynamic lung compliance (Cdyn), mechanical power (MP), and MP normalized to predicted body weight (WMP) between pre-ICU aspirin exposure group and non-aspirin exposure group for different time periods.

|  | **NLR** | | **RDW** | | **P/F** | | **Cdyn** | | **MP** | | **WMP** | |
| --- | --- | --- | --- | --- | --- | --- | --- | --- | --- | --- | --- | --- |
|  | ***Median(IQR)*** | ***P*** | ***Median(IQR)*** | ***P*** | ***Median(IQR)*** | ***P*** | ***Median(IQR)*** | ***P*** | ***Median(IQR)*** | ***P*** | ***Median(IQR)*** | ***P*** |
| D1/T1(aspirin) | 7.2(3.7, 11.2) | **0.003** | 15.2(14.2, 16.4) | 0.15 | 81.4(59.0, 112.0) | 0.50 | 98.3(70.6, 128.7) | **0.02** | 14.1(11.3, 18.8) | **0.045** | 219.6(167.9, 288.9) | **0.02** |
| D1/T1(non-aspirin) | 11.0(6.4, 17.5) |  | 15.6(14.0, 17.7) |  | 92.3(65.0, 117.0) |  | 87.5(65, 113.6) |  | 16.3(11.8, 23.5) |  | 262.6(178.1, 356.3) |  |
| D2/T2(aspirin) | 15.0(9.0, 17.0) | 0.77 | 15.3(14.5, 16.5) | 0.28 | 157.5(95.6, 224.5) | 0.17 | 110.6(82.0, 141.3) | **0.01** | 14.4(11.6, 19.2) | **0.004** | 229.2(172.6, 286.6) | **0.003** |
| D2/T2(non-aspirin) | 12.9(10.4, 22.5) |  | 15.6(14.3, 17.5) |  | 122.0(90.0, 198.0) |  | 89.8(68.7, 123.1) |  | 18.2(13.2, 25.3) |  | 289.8(207.1, 374.7) |  |
| D3/T3(aspirin) | 12.8(8.2, 19.0) | 0.71 | 15.3(14.6, 16.5) | 0.13 | 162.0(103.7, 212.0) | 0.67 | 107.0(67.9, 143.5) | 0.32 | 15.9(12.9, 21.3) | 0.19 | 238.7(207.0, 301.4) | 0.06 |
| D3/T3(non-aspirin) | 11.0(7.1, 17.6) |  | 15.7(14.5, 17.4) |  | 147.8(109.6, 220.0) |  | 89.6(66.9, 127.7) |  | 17.7(12.4, 26.7) |  | 286.7(216.8, 387.3) |  |
| D4/T4(aspirin) | 9.4(6.6, 12.1) | 0.78 | 15.4(14.4, 16.4) | **0.04** | 157.6(110.0, 209.5) | 0.79 | 109.9(82.2, 167.7) | 0.10 | 16.7(11.9, 21.8) | 0.31 | 273.4(202.0, 300.6) | 0.35 |
| D4/T4(non-aspirin) | 10.3(5.8, 16.4) |  | 15.9(14.7, 17.4) |  | 146.7(110.0, 227.5) |  | 82.6(74.8, 134.3) |  | 19.1(12.8, 25.3) |  | 264.8(21.08, 359.3) |  |
| D5/T5(aspirin) | 8.3(5.3, 12.8) | 0.95 | 15.6(14.4, 16.7) | **0.04** | 160.0(125.0, 235.0) | 0.36 | 124.1(89.6, 178.4) | **0.02** | 14.7(13.2, 21.5) | **0.046** | 235.4(183.2, 290.3) | **0.049** |
| D5/T5(non-aspirin) | 8.3(4.5, 18.3) |  | 15.9(14.8, 17.4) |  | 140.2(106.6, 220.9) |  | 94.4(71.8, 141.2) |  | 19.3(13.5, 25.7) |  | 277.0(200.8, 371.0) |  |
| T6(aspirin) | - | - | - | - | 188.0(143.4, 264.0) | **0.04** | 124.3(85.8, 167.1) | **0.05** | 14.8(11.9, 19.5) | **0.04** | 233.7(165.3, 272.9) | **0.03** |
| T6(non-aspirin) | - |  | - |  | 138.0(100.6, 239.4) |  | 100.3(76.0, 144.87) |  | 18.1(13.5, 24.8) |  | 279.2(195.6, 371.0) |  |

D1-D5 represent the 1^st^, 2^nd^, 3^rd^, 4^th^, and 5^th^ day after ICU admission, while the T1-T6 represent the 12^th^, 24^th^, 36^th^, 48^th^, 60^th^, and 72^th^ hour after initiation of invasive mechanical ventilation;

NLR: neutrophil to lymphocyte ratio; RDW: red cell distribution width; PF: [oxygenation index](javascript:;); Cdyn: pulmonary dynamic compliance; WMP: mechanical power normalized to predicted body weight.

Table S7: The mediation regression analysis in the association between pre-ICU aspirin exposure and 28-day mortality, 60-day mortality and hospital mortality.

| **Outcome: 28-day mortality** | **Coeff** | **SE** | **95% CI** | ***P*** |
| --- | --- | --- | --- | --- |
| Aspirin (Yes vs No) → TWA_NLR | -4.59 | 1.66 | -7.87, -1.32 | **0.006** |
| Aspirin (Yes vs No) → TWA_RDW | -0.58 | 0.35 | -1.27, 0.10 | 0.094 |
| Aspirin (Yes vs No) → TWA_PF | 23.08 | 16.73 | -10.00, 56.16 | 0.170 |
| Aspirin (Yes vs No) → TWA_Cdyn | 20.19 | 7.71 | 4.95, 35.42 | **0.01** |
| Aspirin (Yes vs No) → TWA_WMP | -51.74 | 19.00 | -89.32, -14.17 | **0.007** |
| Aspirin (Yes vs No) → Outcome | -0.73 | 0.45 | -1.60, -0.13 | **0.01** |
| TWA_NLR→ Outcome | 0.03 | 0.02 | 0.018, 0.07 | **0.038** |
| TWA_RDW → Outcome | 0.28 | 0.10 | -0.06, 0.47 | 0.120 |
| TWA_PF → Outcome | 0.001 | 0.005 | -0.004, 0.005 | 0.799 |
| TWA_Cdyn → Outcome | -0.02 | 0.005 | -0.032, -0.01 | **0.041** |
| TWA_WMP → Outcome | 0.004 | 0.002 | 0.003, 0.008 | **0.033** |
| **Outcome: Hospital mortality** |  |  |  |  |
| Aspirin (Yes vs No) → TWA_NLR | -4.59 | 1.66 | -7.87, -1.32 | **0.006** |
| Aspirin (Yes vs No) → TWA_RDW | -0.58 | 0.35 | -1.27, 0.10 | 0.094 |
| Aspirin (Yes vs No) → TWA_PF | 23.08 | 16.73 | -10.00, 56.16 | 0.170 |
| Aspirin (Yes vs No) → TWA_Cdyn | 20.19 | 7.71 | 4.95, 35.42 | **0.01** |
| Aspirin (Yes vs No) → TWA_WMP | -51.74 | 19.00 | -89.32, -14.17 | **0.007** |
| Aspirin (Yes vs No) → Outcome | -0.65 | 0.46 | -1.41, -0.38 | **0.026** |
| TWA_NLR→ Outcome | 0.02 | 0.02 | 0.006, 0.08 | **0.043** |
| TWA_RDW → Outcome | 0.24 | 0.11 | -0.03, 0.45 | 0.26 |
| TWA_PF → Outcome | 0.001 | 0.002 | -0.003, 0.005 | 0.544 |
| TWA_Cdyn → Outcome | -0.016 | 0.004 | -0.022, -0.0004 | **0.047** |
| TWA_WMP → Outcome | 0.004 | 0.002 | 0.002, 0.009 | **0.009** |
| **Outcome: 60-day mortality** |  |  |  |  |
| Aspirin (Yes vs No) → TWA_NLR | -4.59 | 1.66 | -7.87, -1.32 | **0.006** |
| Aspirin (Yes vs No) → TWA_RDW | -0.58 | 0.35 | -1.27, 0.10 | 0.094 |
| Aspirin (Yes vs No) → TWA_PF | 23.08 | 16.73 | -10.00, 56.16 | 0.170 |
| Aspirin (Yes vs No) → TWA_Cdyn | 20.19 | 7.71 | 4.95, 35.42 | **0.01** |
| Aspirin (Yes vs No) → TWA_WMP | -51.74 | 19.00 | -89.32, -14.17 | **0.007** |
| Aspirin (Yes vs No) → Outcome | -0.81 | 0.43 | -1.75, -0.42 | **0.003** |
| TWA_NLR→ Outcome | 0.03 | 0.02 | 0.012, 0.072 | **0.044** |
| TWA_RDW → Outcome | 0.15 | 0.10 | -0.02, 0.40 | 0.174 |
| TWA_PF → Outcome | 0.003 | 0.002 | -0.002, 0.007 | 0.801 |
| TWA_Cdyn → Outcome | -0.012 | 0.003 | -0.02, 0.005 | 0.80 |
| TWA_WMP → Outcome | 0.002 | 0.002 | -0.003, 0.010 | 0.21 |

TWA: time weighted average; NLR: neutrophil to lymphocyte ratio; RDW: red cell distribution width; PF: [oxygenation index](javascript:;); Cdyn: pulmonary dynamic compliance; WMP: mechanical power normalized to predicted body weight.


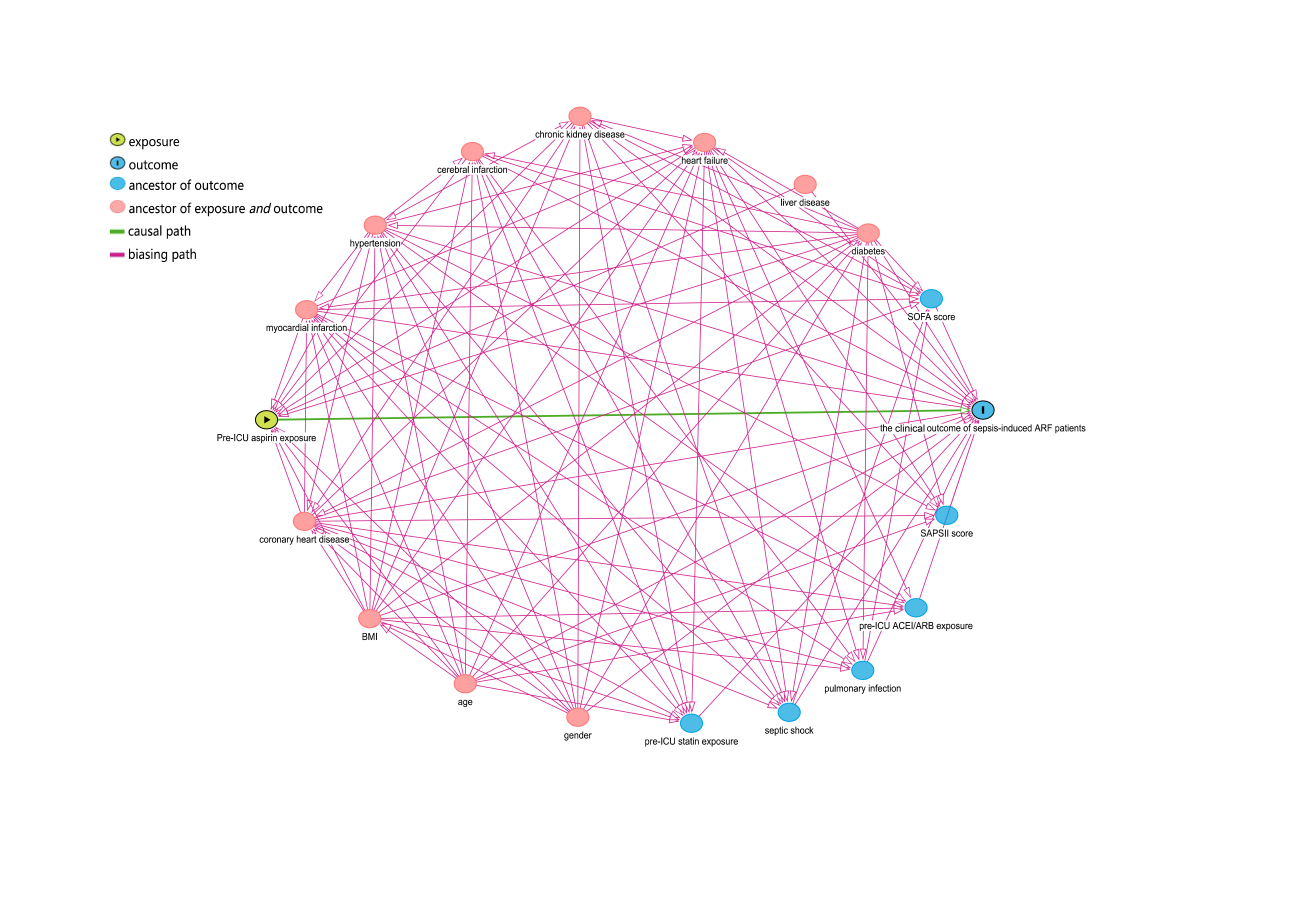


Figure S1: Directed acyclic graph of the association between pre-ICU aspirin exposure and the mortality risk of patients with sepsis-associated acute respiratory failure.


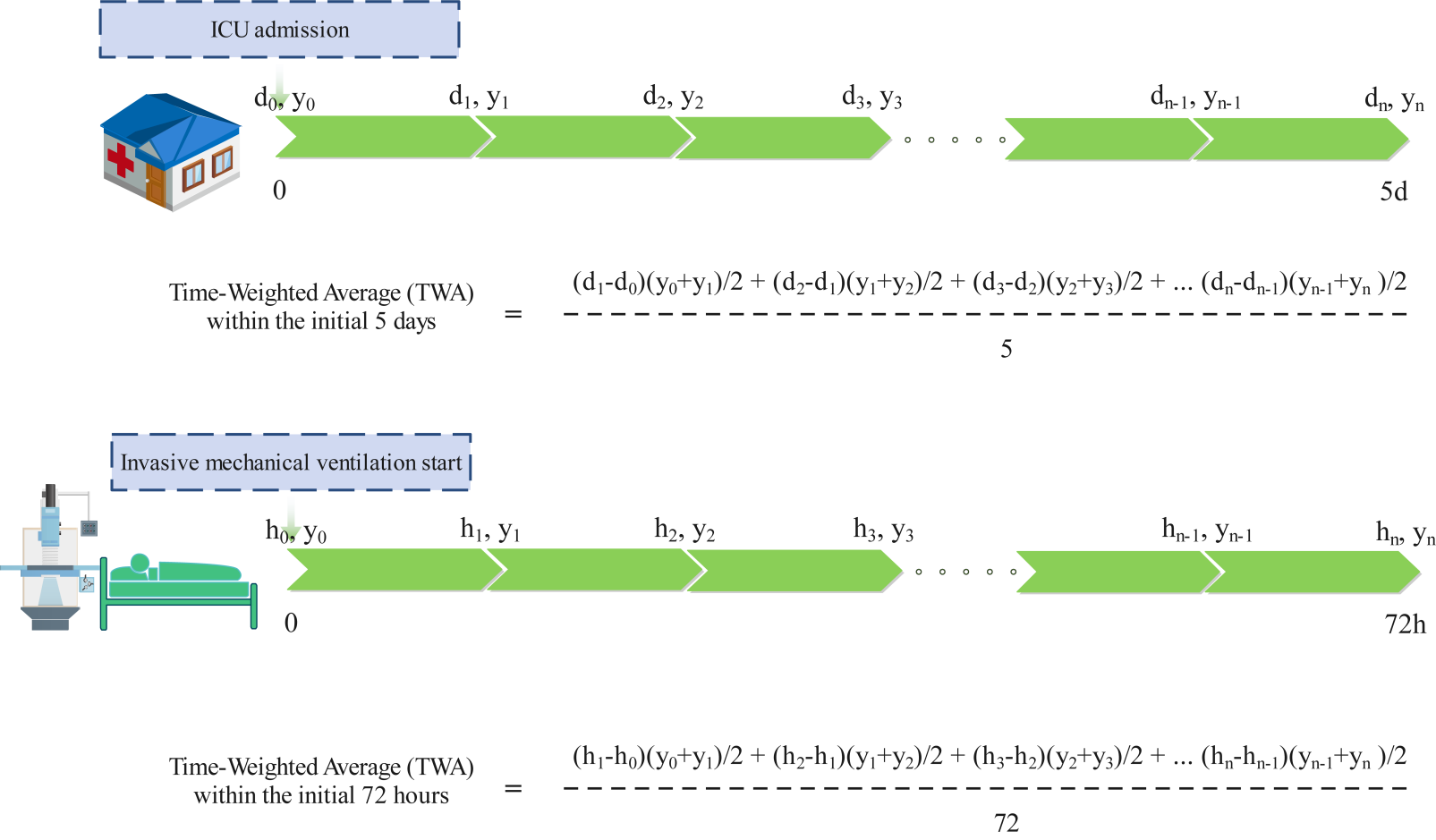


Figure S2: Calculation of time-weighted average red cell distribution width, neutrophil to lymphocyte ratio, PaO_2_/FiO_2,_ dynamic lung compliance, and mechanical power normalized to predicted body weight extracted from MIMIC-IV (2.0) databases.


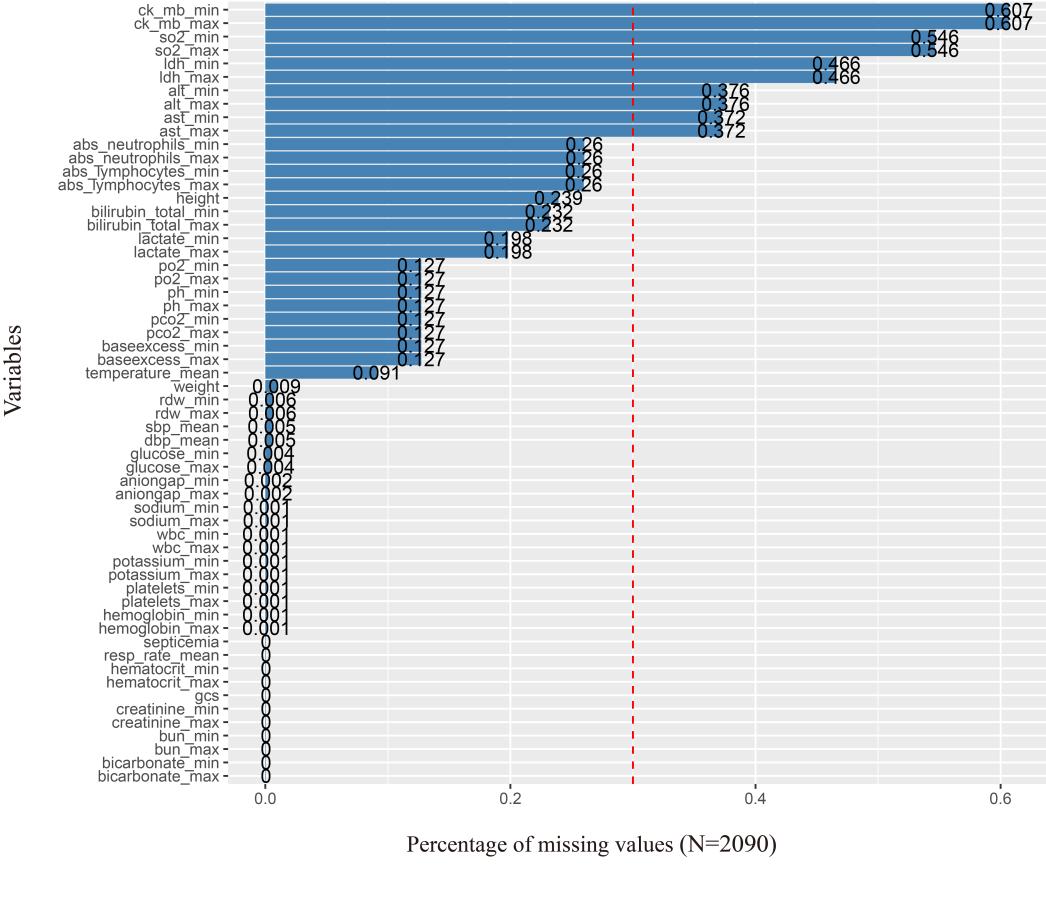


Figure S3: The percentage of missing data in included patients, and the variables with missing rate > 30% were excluded.


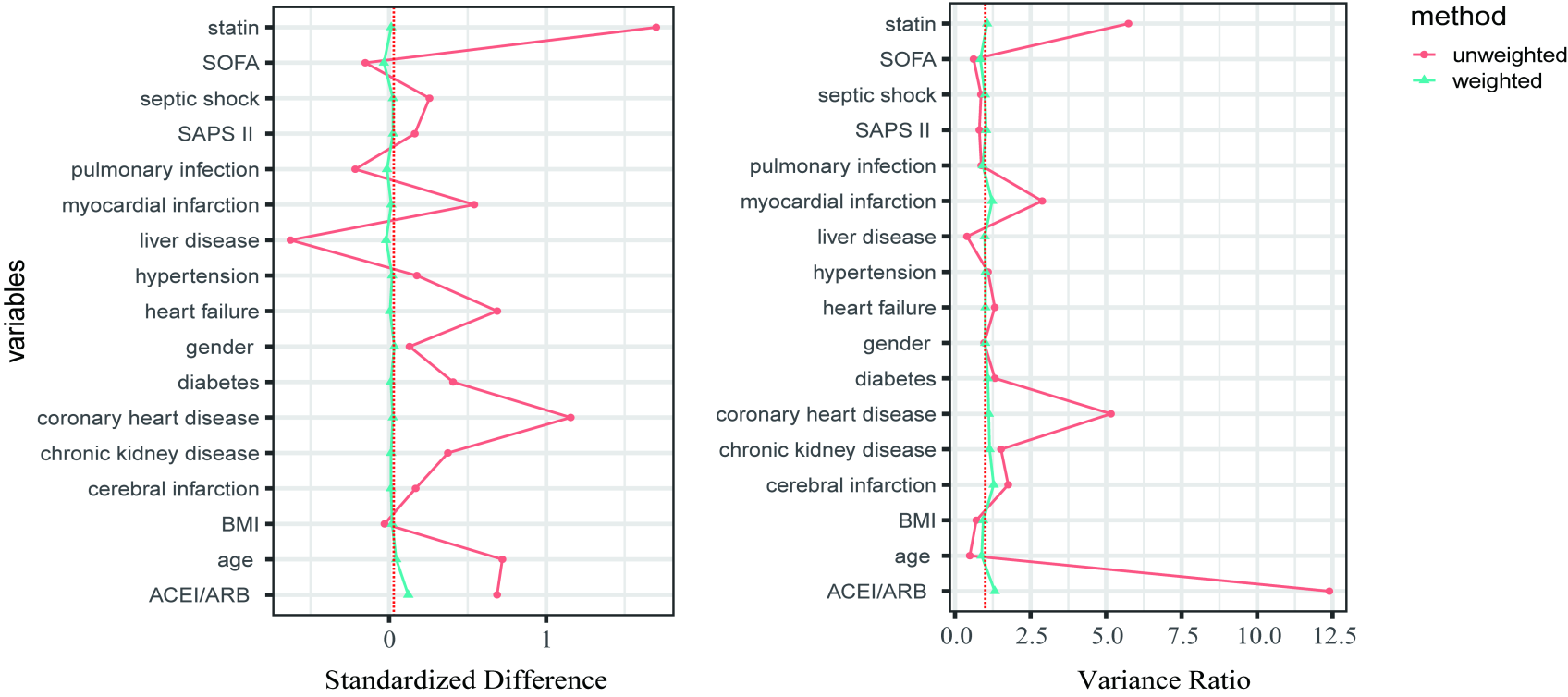


Figure S4: The standardized differences and variance ratios before and after augmented inverse propensity weighted.


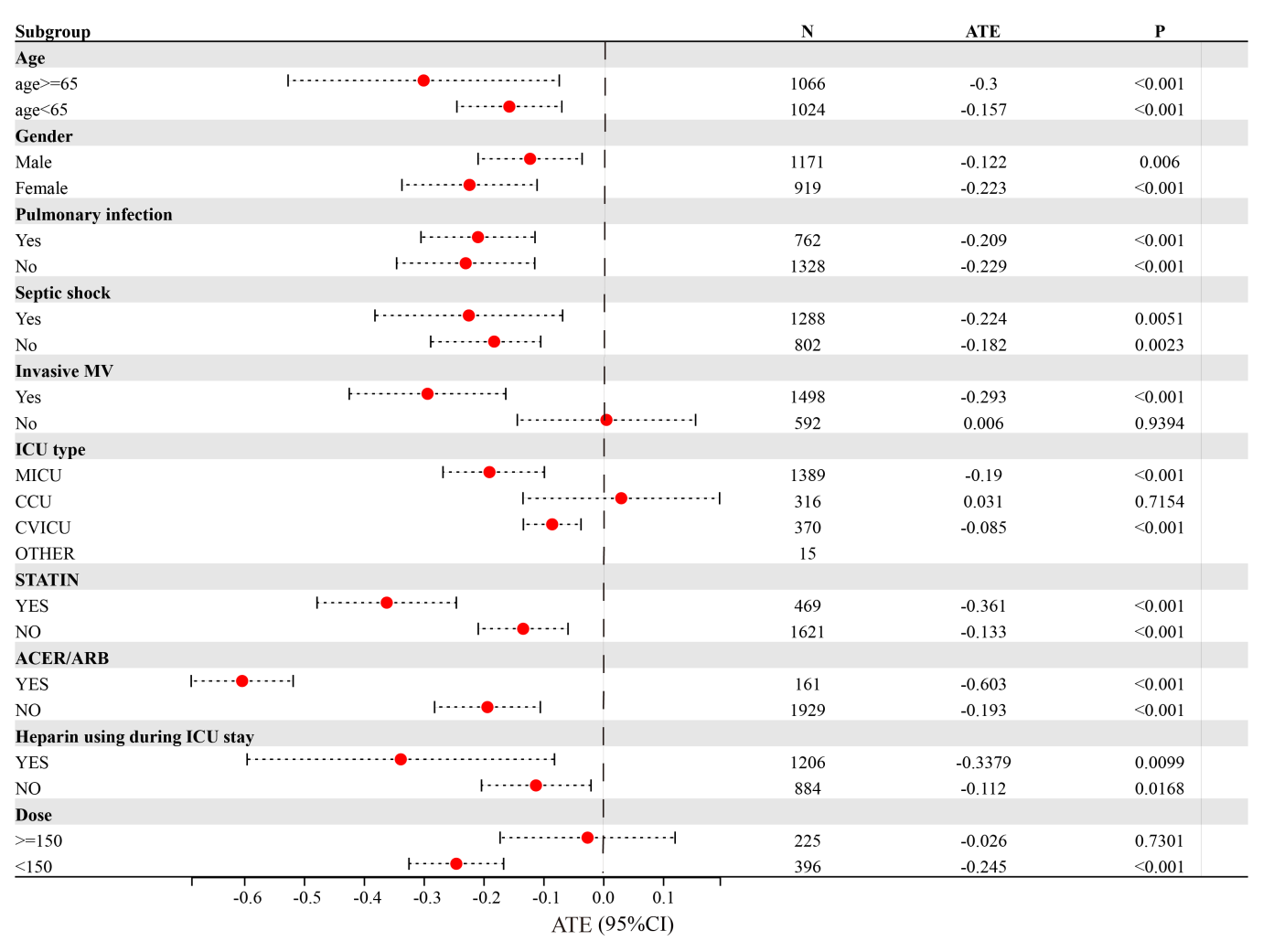


Figure S5: AIPW analysis of causal effect of pre-ICU aspirin on 28-day mortality stratified by age, gender, infection site, septic shock or not, received invasive mechanical ventilation or not, ICU type, pre-ICU statin exposure or not, pre-ICU ACEI/ARB exposure, and the initial aspirin dose.


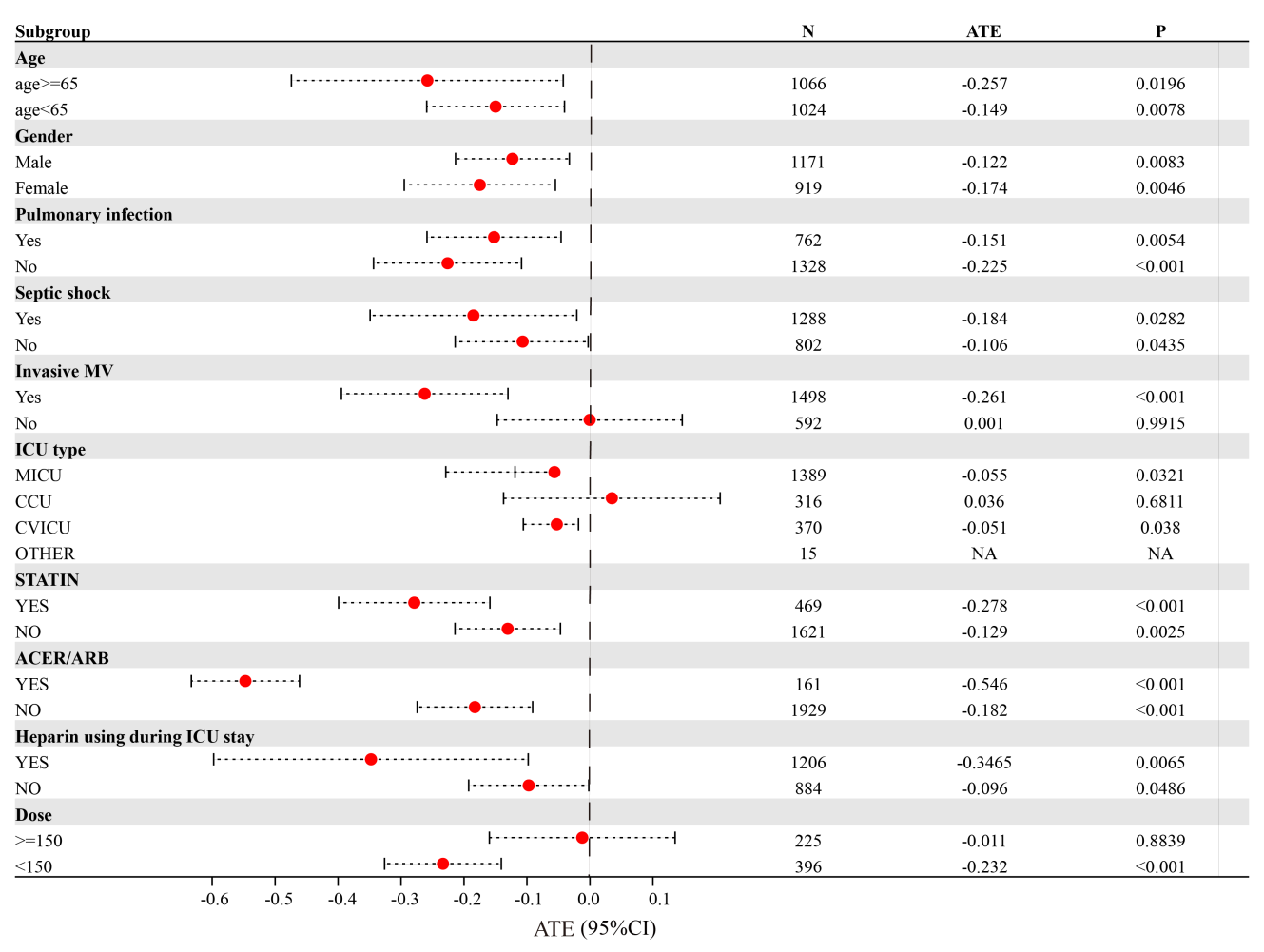


Figure S6: AIPW analysis of causal effect of pre-ICU aspirin on 60-day mortality stratified by age, gender, infection site, septic shock or not, received invasive mechanical ventilation or not, ICU type, pre-ICU statin exposure or not, pre-ICU ACEI/ARB exposure, and the initial aspirin dose.


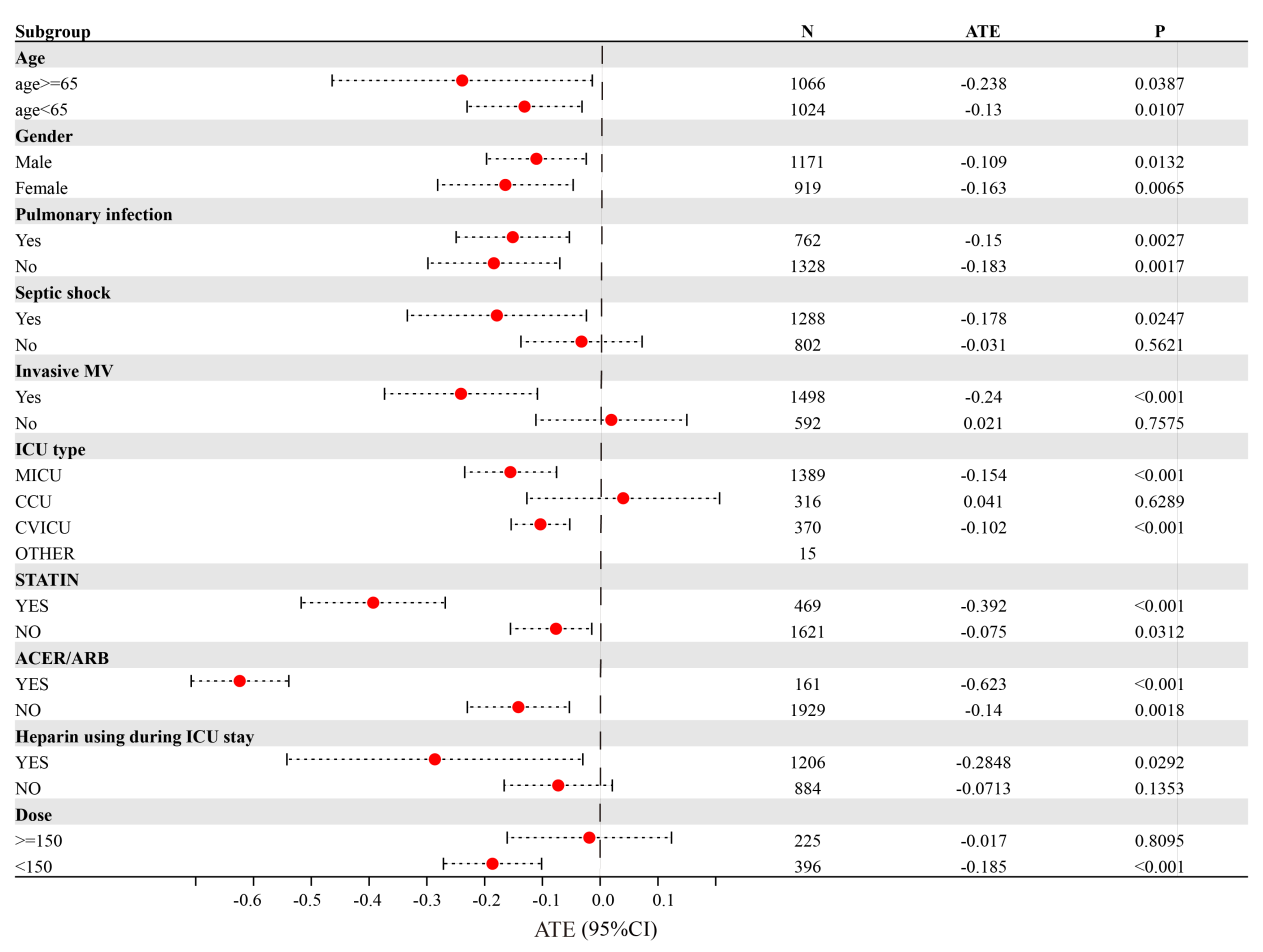


Figure S7: AIPW analysis of causal effect of pre-ICU aspirin on hospital mortality stratified by age, gender, infection site, septic shock or not, received invasive mechanical ventilation or not, ICU type, pre-ICU statin exposure or not, pre-ICU ACEI/ARB exposure, and the initial aspirin dose.


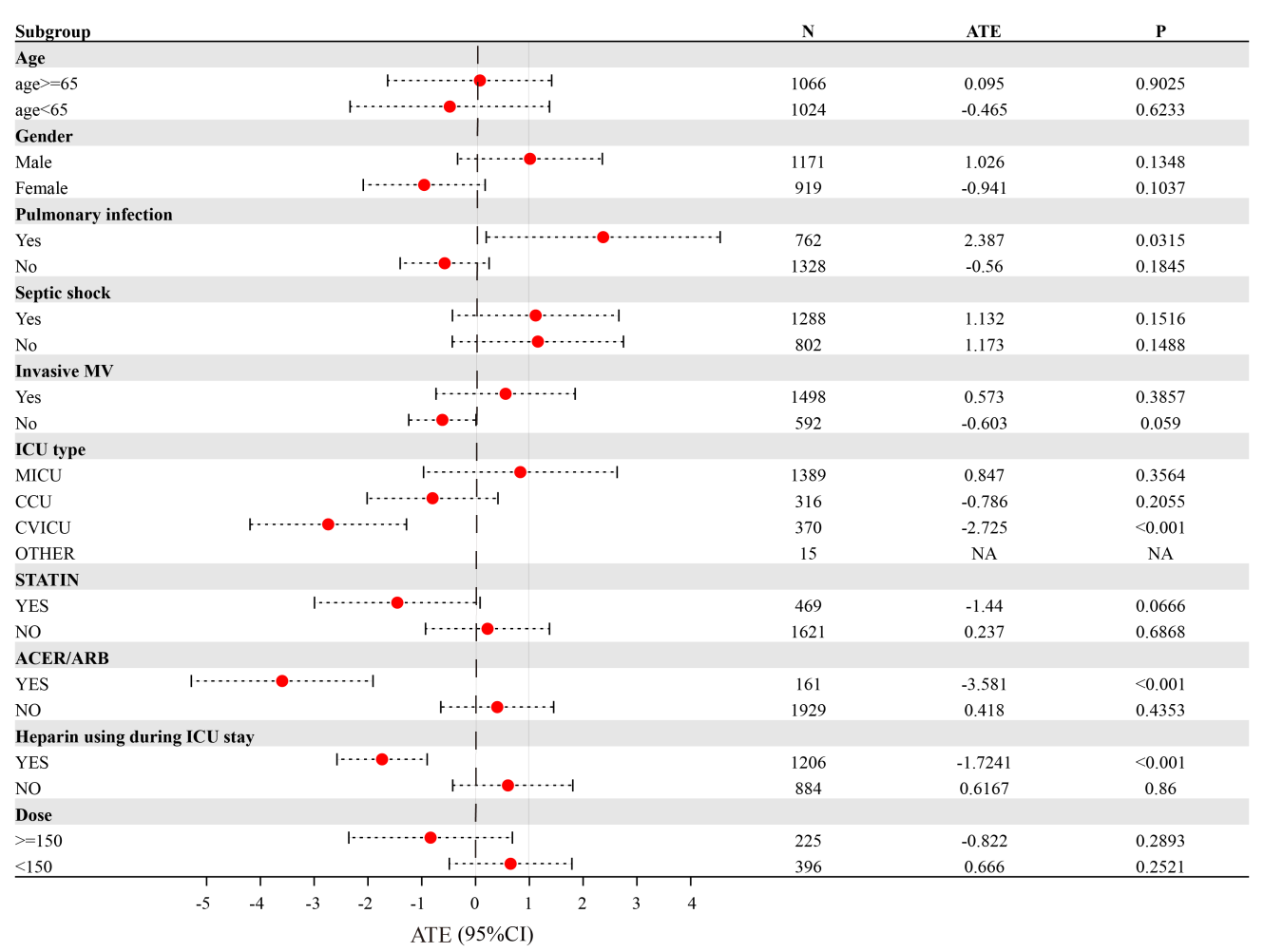


Figure S8: AIPW analysis of causal effect of pre-ICU aspirin on ICU length of stay stratified by age, gender, infection site, septic shock or not, received invasive mechanical ventilation or not, ICU type, pre-ICU statin exposure or not, pre-ICU ACEI/ARB exposure, and the initial aspirin dose.


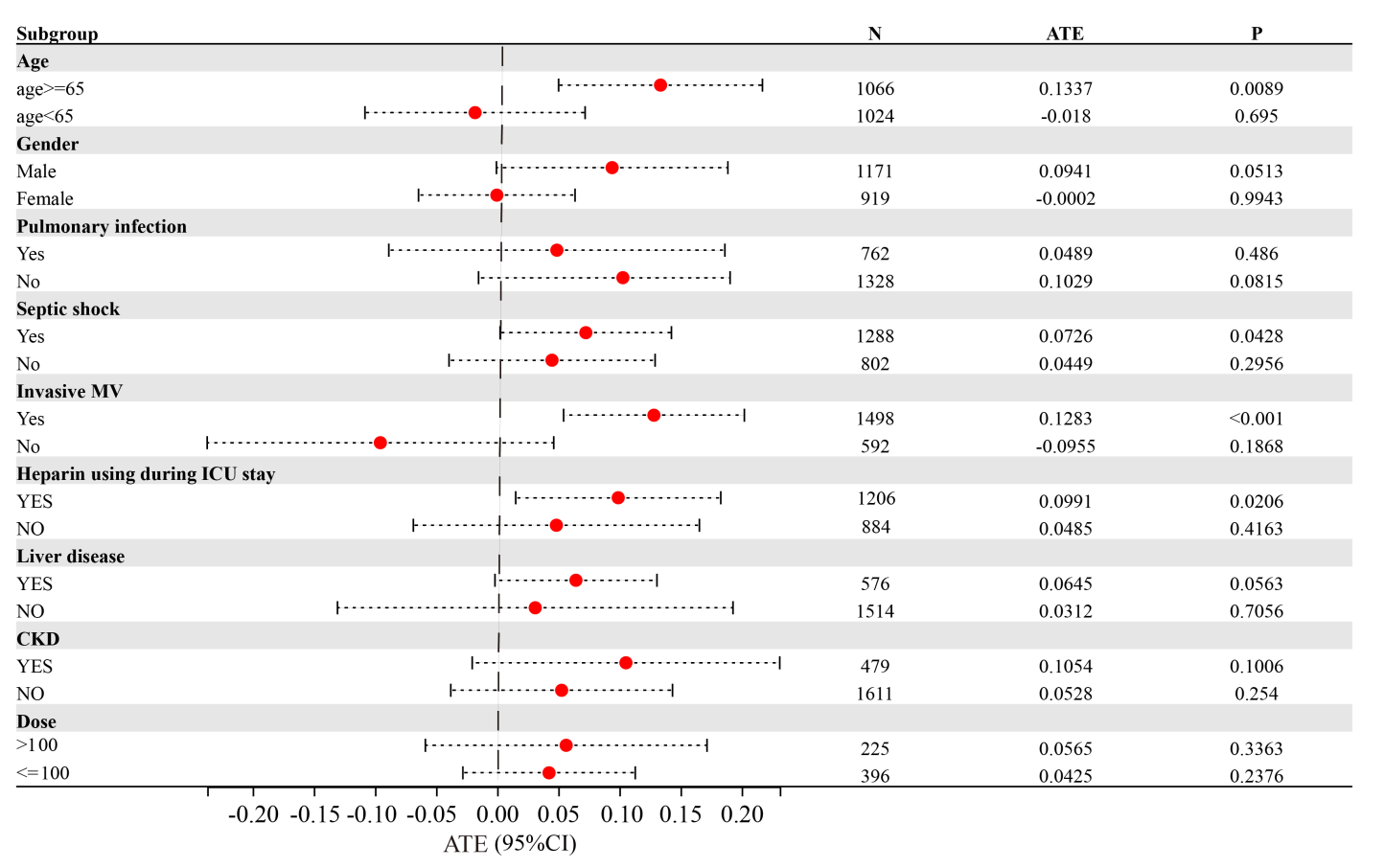


Figure S9: AIPW analysis of causal effect of pre-ICU aspirin on thrombocytopenia risk stratified by age, gender, infection site, septic shock or not, received invasive mechanical ventilation or not, received heparin during ICU stay or not, combined with liver disease and [chronic renal failure](javascript:;) or not, and the initial aspirin dose.


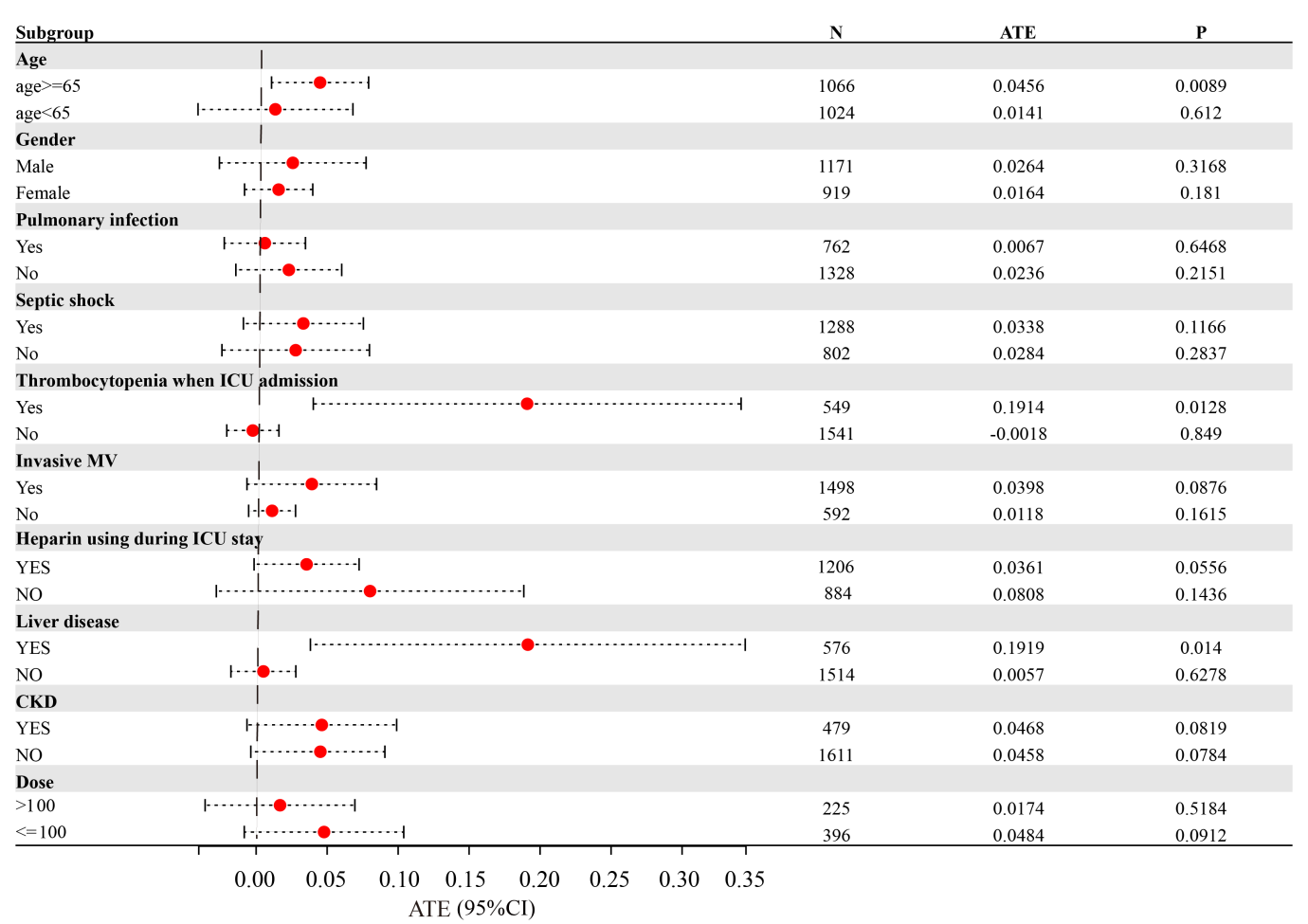


Figure S10: AIPW analysis of causal effect of pre-ICU aspirin on major gastrointestinal hemorrhage risk stratified by age, gender, infection site, septic shock or not, thrombocytopenia on ICU admission, received invasive mechanical ventilation or not, received heparin during ICU stay or not, combined with liver disease and [chronic renal failure](javascript:;) or not, and the initial aspirin dose.


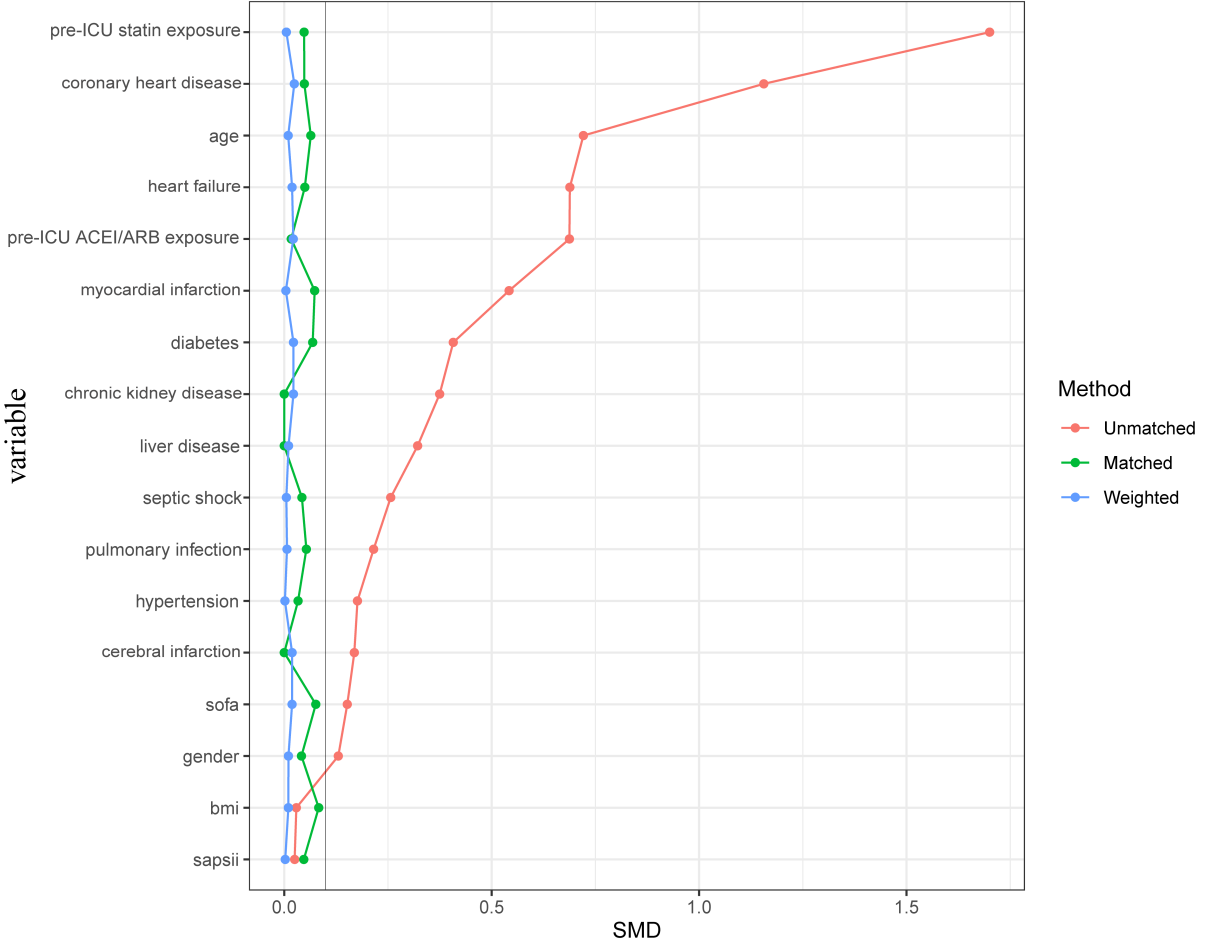


Figure S11: The standardized mean difference after propensity score matching.


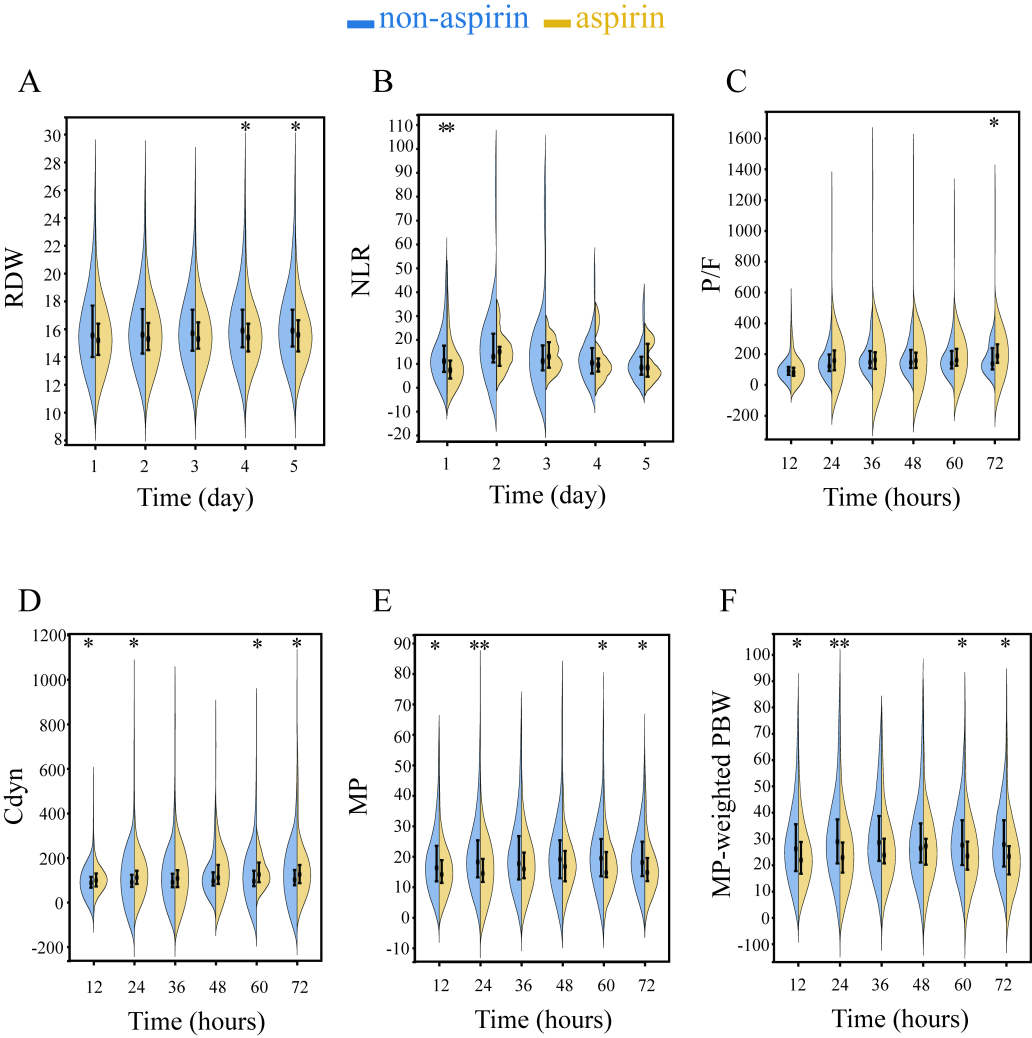


Figure S12: Violin plot of red cell distribution width (RDW), neutrophil to lymphocyte ratio (NLR), PaO_2_/FiO_2_ (P/F), dynamic lung compliance (Cdyn), mechanical power (MP), and MP normalized to predicted body weight (WMP) for different time periods; A-C: NLR and RDW were measured within 5 days after ICU admission; P/F, Cdyn, MP, and WMP were measured within 72 hours after initiation of invasive mechanical ventilation; * *P* <0.05, ** *P* < 0.01.


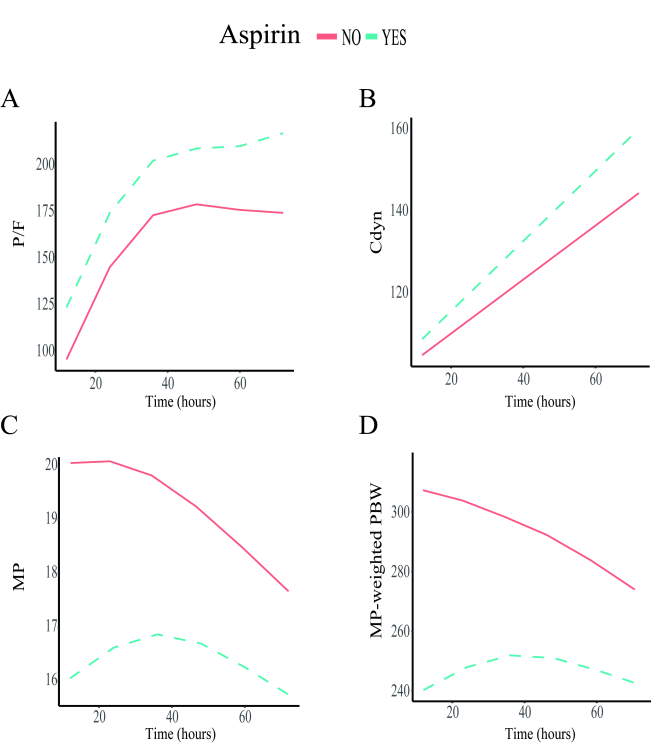


Figure S13: Association between PaO_2_/FiO_2_ (P/F), dynamic lung compliance (Cdyn), mechanical power (MP), MP normalized to predicted body weight (WMP) and low-dose aspirin exposure using the generalized additive mixed model after adjusting the initial red cell distribution width (RDW) and neutrophil to lymphocyte ratio (NLR) levels at the initiation of invasive mechanical ventilation. A: P/F of the low-dose aspirin exposure group increased by 0.51 mmHg (SE=0.16, *P*=0.012) every hour compared to that of the non-exposure group within 72 hours after receiving invasive mechanical ventilation; B: Cdyn of the low-dose exposure group increased by 0.27 ml/cmH_2_0 (SE=0.13, *P*=0.039) every hour compared to that of the non-exposure group within 72 hours after receiving invasive mechanical ventilation; C-D: MP (-3.14J/min, SE=1.30, *P*<0.001) and WMP levels (-52.24×10^−3^ J/min/kg, SE=20.11, *P*=0.0104) of the low-dose aspirin exposure group are significantly lower than that of the non-exposure group within the initial 12 hours after receiving invasive mechanical ventilation.


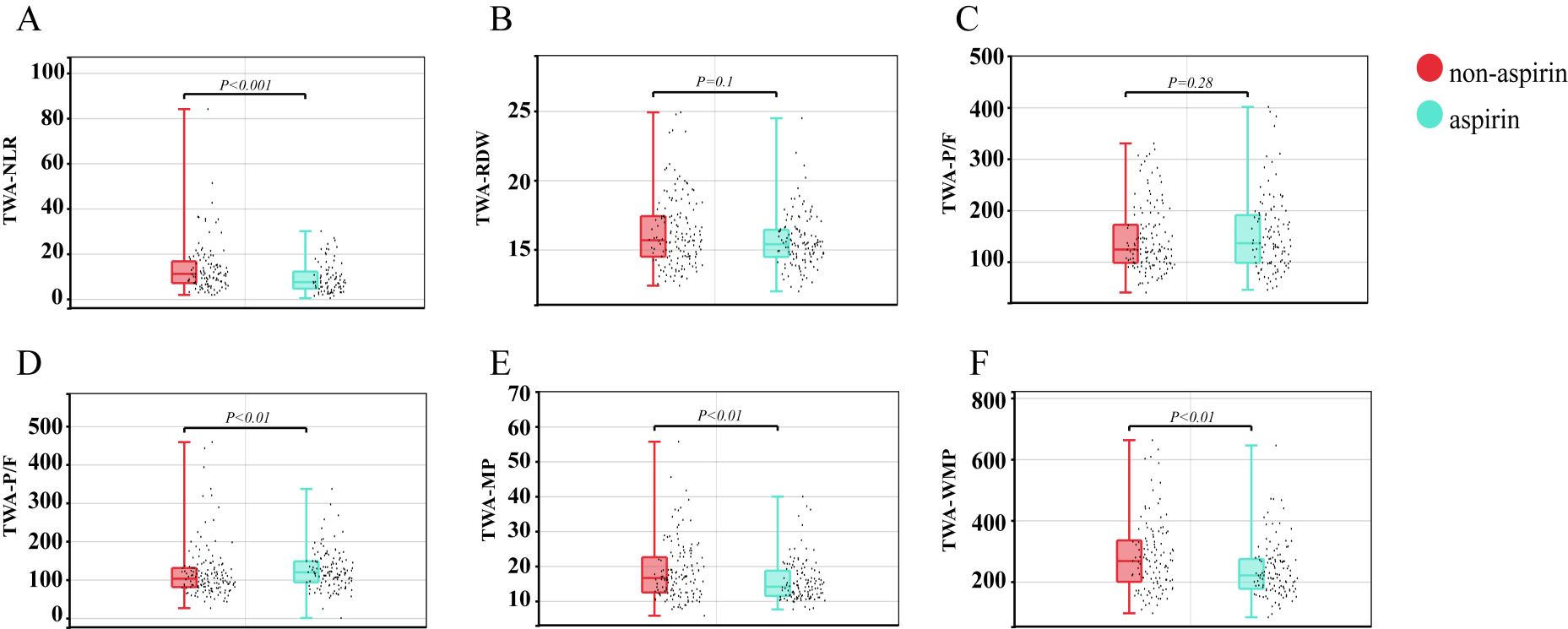


Figure S14: Boxplot graphf of time-weighted average red cell distribution width (TWA-RDW), time-weighted average neutrophil to lymphocyte ratio (TWA-NLR), time-weighted average PaO_2_/FiO_2_ (TWA-P/F), time-weighted average dynamic lung compliance (TWA-Cdyn), time-weighted average mechanical power (TWA-MP), and time-weighted average MP normalized to predicted body weight (TWA-WMP).


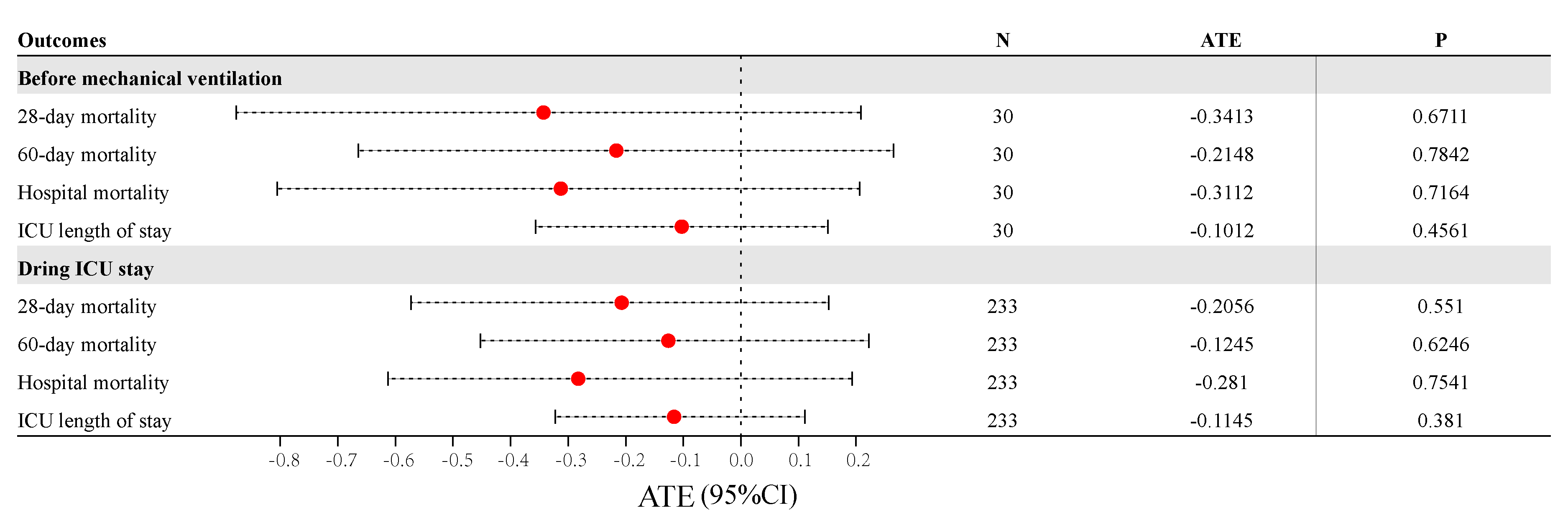


Figure S15: AIPW analysis of causal effect of aspirin used before mechanical ventilation or during the ICU stay on the 28-day mortality, 60-day mortality, hospital mortality, and ICU length of stay when comparing with those who did not.
